# Genotype–Phenotype Association Analysis Reveals New Pathogenic Factors for Osteogenesis Imperfecta Disease

**DOI:** 10.3389/fphar.2019.01200

**Published:** 2019-10-15

**Authors:** Jingru Shi, Meng Ren, Jinmeng Jia, Muxue Tang, Yongli Guo, Xin Ni, Tieliu Shi

**Affiliations:** ^1^Center for Bioinformatics and Computational Biology, and the Institute of Biomedical Sciences, School of Life Sciences, East China Normal University, Shanghai, China; ^2^Big Data and Engineering Research Center, Beijing Key Laboratory for Pediatric Diseases of Otolaryngology, Head and Neck Surgery, MOE Key Laboratory of Major Diseases in Children, Beijing Children’s Hospital, National Center for Children’s Health, Beijing Pediatric Research Institute, Capital Medical University, Beijing, China; ^3^Biobank for Clinical Data and Samples in Pediatrics, Beijing Children’s Hospital, National Center for Children’s Health, Beijing Pediatric Research Institute, Capital Medical University, Beijing, China; ^4^Department of Otolaryngology, Head and Neck Surgery, Beijing Children’s Hospital, National Center for Children’s Health, Capital Medical University, Beijing, China

**Keywords:** osteogenesis imperfecta, genotype, phenotype, novel candidate pathogenic genes, novel candidate pathogenic variations

## Abstract

Osteogenesis imperfecta (OI), mainly caused by structural abnormalities of type I collagen, is a hereditary rare disease characterized by increased bone fragility and reduced bone mass. Clinical manifestations of OI mostly include multiple repeated bone fractures, thin skin, blue sclera, hearing loss, cardiovascular and pulmonary system abnormalities, triangular face, dentinogenesis imperfecta (DI), and walking with assistance. Currently, 20 causative genes with 18 subtypes have been identified for OI, of them, variations in *COL1A1* and *COL1A2* have been demonstrated to be major causative factors to OI. However, the complexity of the bone formation process indicates that there are potential new pathogenic genes associated with OI. To comprehensively explore the underlying mechanism of OI, we conducted association analysis between genotypes and phenotypes of OI diseases and found that mutations in *COL1A1* and *COL1A2* contributed to a large proportion of the disease phenotypes. We categorized the clinical phenotypes and the genotypes based on the variation types for those 155 OI patients collected from literature, and association study revealed that three phenotypes (bone deformity, DI, walking with assistance) were enriched in two variation types (the Gly-substitution missense and groups of frameshift, nonsense, and splicing variations). We also identified four novel variations (c.G3290A (p.G1097D), c.G3289C (p.G1097R), c.G3289A (p.G1097S), c.G3281A (p.G1094D)) in gene *COL1A1* and two novel variations (c.G2332T (p.G778C), c.G2341T (p.G781C)) in gene *COL1A2*, which could potentially contribute to the disease. In addition, we identified several new potential pathogenic genes (*ADAMTS2*, *COL5A2*, *COL8A1*) based on the integration of protein–protein interaction and pathway enrichment analysis. Our study provides new insights into the association between genotypes and phenotypes of OI and novel information for dissecting the underlying mechanism of the disease.

## Introduction

Osteogenesis imperfecta (OI) is a phenotypically and genetically heterogeneous group of bone disorders characterized by bone fragility and skeletal deformity, owing to the abnormality of type I collagen formed by two α1(I) chains (encoded by *COL1A1* gene) and one α2(I) chain (encoded by *COL1A2* gene). Individuals with OI have low bone mass, which results in deformity of long bones, vertebral anomalies and fractures, shortening of extremities, and skull defect (Marini et al., 2007). The observed extra-skeletal phenotypes include dentinogenesis imperfecta (DI), thin skin, blue sclera, scoliosis, cardiovascular and pulmonary system abnormalities, triangular face, and hearing impairment (Foster et al., 2014; Marini et al., 2017). Previous studies categorize OI into four subtypes (types I–IV) based on clinical findings, inheritance patterns, and radiographic features: OI type I is the mildest form, OI type II is the perinatal lethal form, while OI type III is the most severe form, and OI type IV is characterized by the mild to moderate form (Sillence et al., 1979; Rauch et al., 2010; Lin et al., 2015; Mrosk et al., 2018). With an in-depth understanding of OI disease, more subtypes have been defined and added into OI’s original classification system, making the number of subtypes updated to 18 (Forlino and Marini, 2016; Marini et al., 2017; Lu et al., 2019).

Current evidences demonstrate that *COL1A1* and *COL1A2* are the main factors in the cause of OI, as approximately 85% to 90% of cases are disturbed by them, and all of the four subtypes are involved in *COL1A1* and *COL1A2* genes (http://www.le.ac.uk/ge/collagen/). There are two general categories of mutational defects occurred in *COL1A1*/*COL1A2*. The first is missense mutation, mainly involving glycine replacement within the Gly-Xaa-Yaa repeat (the Gly-substitution missense), which results in the synthesis of collagen with abnormal structure (Lin et al., 2015). The second is a group of variations that include frameshift, nonsense, and splicing mutations, which mainly lead to the reduced amount of normal type I collagen. Previous studies have shown that the second variation group is often associated with milder phenotypes, while the Gly-substitution missense usually lead to more severe phenotypes (Rauch et al., 2010; Zhang et al., 2012). Considering the phenotypic specificity of the Gly-substitution missense, we would like to investigate more potentially pathogenic Gly-substitution mutations for OI mechanism exploration.

In addition to the confirmed OI-related collagen genes (*COL1A1* and *COL1A2*), in the past decade, a series of studies have found that a set of new non-collagen gene defects affect normal post-translational processing, molecular folding of type I collagen, fibril formation, osteoblast differentiation, and mineralization, leading to rare autosomal recessive, dominant, and X-linked forms of OI (Bregou Bourgeois et al., 2016; Lindert et al., 2016; Marom et al., 2016; Marini et al., 2017). With the rapid development of next-generation sequencing technology, almost 18 pathogenic non-collagen genes have gradually been identified (Forlino and Marini, 2016; Marini et al., 2017; Mrosk et al., 2018), including *BMP1*, *CRTAP*, *P3H1*, *PPIB*, *TMEM38B*, *SERPINH1*, *FKBP10*, *PLOD2*, *IFITM5*, *SERPINF1*, *WNT1*, *CREB3L1*, *SP7*, *SPARC*, *MBTPS2*, *P4HB*, *PLS3,* and *SEC24D*. Based on the complexity of bone formation and clinical observation, we believe that new potential disease-related genes remain to be identified.

Genotype and phenotype associations can provide new insights into understanding the disease mechanism (Geng et al., 2017; Li et al., 2017). The phenotypic severity depends not only on the affected gene, but also on the position of the mutation in the gene. To identify new missense mutations associated with OI, in the present study, we firstly collected genotypic and phenotypic information on 155 patients from literature and evaluated the genotype–phenotype associations. Next, we identified a set of disease-associated variations in *COL1A1* and *COL1A2* by integrative analysis with several software designed to predict functional effect of human missense mutations. In addition, considering the fact that each biological function is accomplished by the interactions of multiple proteins, we performed network-based analysis and pathway enrichment analysis to identify novel candidate risk genes potentially contributing to the development of OI. Considering limited availability of the patient size and the complex pathogenesis for OI, our comprehensive analysis could promote better understanding of OI in the clinical diagnose, genetic counseling, and prenatal diagnosis.

## Materials and Methods

### Data Resources

The 20 confirmed OI pathogenic genes and their related reported variant information were extracted from the Osteogenesis Imperfecta Variant Database (OIVD) (e.g., “DNA change,” “mutation effect,” “protein,” “reference,” etc.) (Fokkema et al., 2011). We manually collected 155 OI patient information from published literature, including genes, mutation type, phenotypes, age, and gender.

InWeb_InBioMap is a scored human protein–protein interaction data resource. It is generated by combining interactions from eight protein interaction databases and providing confidence score for each interacting pair and relevance score for every protein. The confidence score represents a lower bound on the probability for the interaction being a true positive, and the relevance score represents a relationship between one protein and others in a specific network. We selected those interactive pairs with both confidence score of 1 and a relevance score of 1 for subsequent study (Li et al., 2017).

We retrieved the expression patterns for those predicted genes and pathogenic genes in different tissues from GTEx database. GTEx is a data resource platform for exploring correlations between genetic variants and gene expression in multiple human tissues (Consortium, 2013).

We standardized the phenotypic description for those 155 patients based on eRAM system (Jia et al., 2018, Ni and Shi (2017)). The collected information of those cases has been stored into eRAM and PedAM (Jia et al., 2018). eRAM is a comprehensively standardized data resource for rare disease by integrating massive text mining results and data from multiple databases. Currently, there are standardized 15,942 rare diseases with corresponding phenotypes recorded in eRAM.

WikiPathways is an open and integrative database, providing biology pathway information (Slenter et al., 2018). WebGestalt supports multiple functional enrichment analysis based on selected different organism, enrichment method and functional databases (Wang et al., 2017). We conducted pathway enrichment analysis based on WikiPathway functional database and only focused on OI-related pathways among those significant ones (*P* < 0.05).

### The Pathogenic Analysis for Variants in OI-Related Genes

To avoid analysis bias resulted from the insufficient reported cases, we only performed the prediction of new pathogenic OI-related variants on *COL1A1* and *COL1A2* genes. All variations information on *COL1A1* and *COL1A2* genes was downloaded from ANNOVAR database (Wang et al., 2010).

ANNOVAR provides functional annotation of single-nucleotide variants (SNVs), insertions, and deletions with more than 10 different tools, including SIFT, CADD, PolyPhen-2, and GERP++. The pathogenic ability of each variation is predicted by those tools with defined cutoff values. SIFT is a tool that uses sequence homology to predict whether a substitution affects protein function and whether amino acid substitutions at specific positions of the protein have phenotypic effects (Ng and Henikoff, 2001). CADD integrates allelic diversity, pathogenicity, functional annotations, and severity of disease, which has the ability to rank known pathogenic mutations by disease severity in individual genome (Kircher et al., 2014). PolyPhen-2 predicts the functional significance of allelic replacement with multiple parameters. We used HumVar-trained results in this tool (Adzhubei et al., 2010). GERP++ recognizes high-resolution regions with nucleotide substitution defects and measures these defects as “rejected substitutions” (Cooper et al., 2005). We used the default cutoff values for each of those four softwares (SIFT_score = 0, Polyphen2_HVAR_score = 1, CADD_phred > 30, GERP++_RS > 5) to define the possible pathogenic ability of each variation on *COL1A1*/*COL1A2* gene.

**Figure 1:**
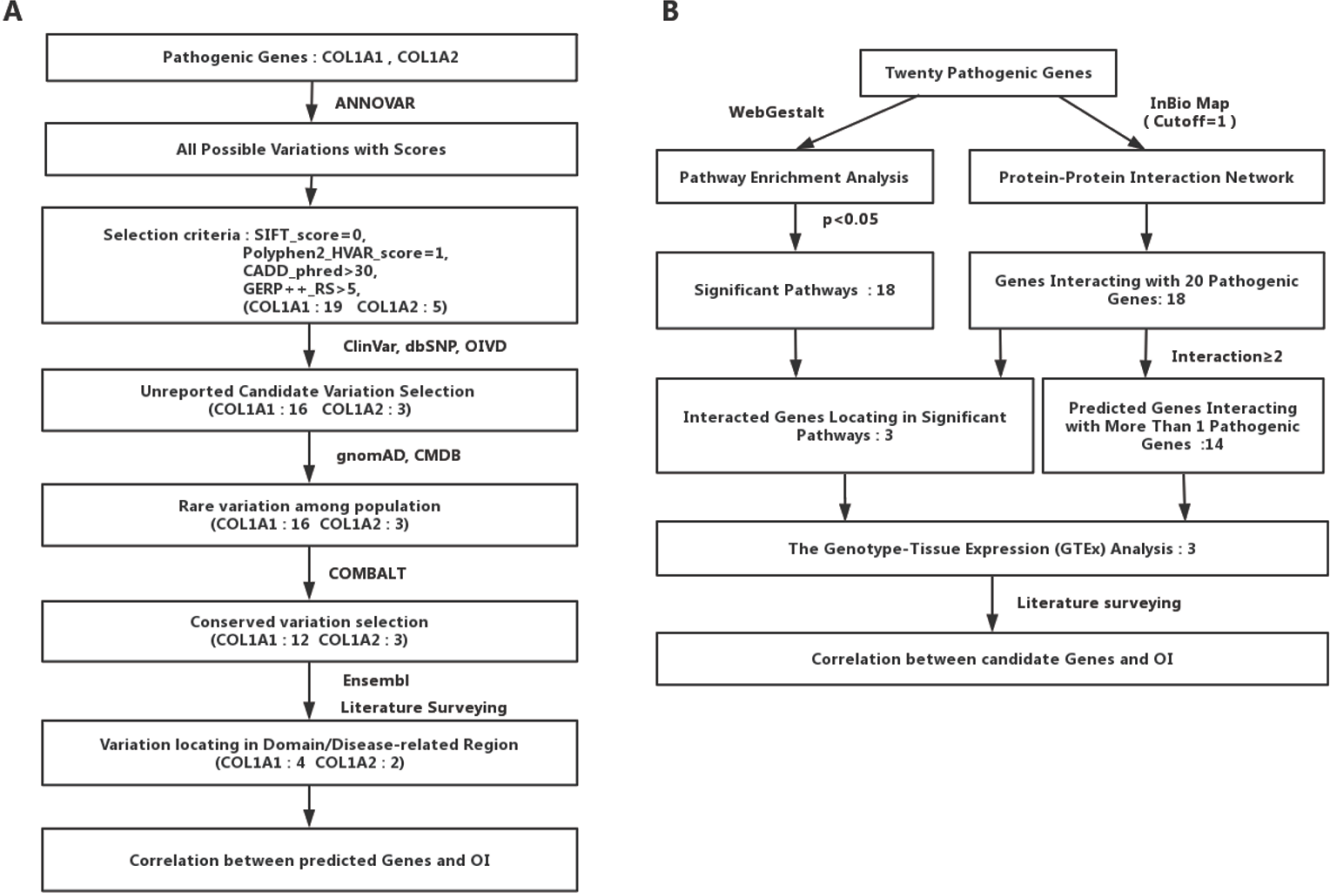
Detailed flow charts for the analysis process. **(A)** A flowchart for OI-related pathogenic variations prediction. **(B)** A flowchart for OI-related genes prediction.

We collected the allele frequency for corresponding variants from gnomAD (Lek et al., 2016) and Chinese Millionome Database (CMDB) (Liu et al., 2018). To further analyze the pathogenicity of those predicted mutations based on the regional conservation, we used COMBALT (Constraint-based Multiple Alignment Tool) to conduct multiple-sequence alignment and identify the conservation for those mutations (Papadopoulos and Agarwala, 2007). At last, we searched the domain information of every predicted mutation by literature surveying and Ensembl genome browser 98 (Cunningham et al., 2019). The whole process was displayed in **Figure 1A**.

### Novel Candidate Risk Genes Identification

To identify potential new risk factor for OI, we first mapped those 20 experimentally confirmed pathogenic genes into InWeb_InBioMap data resource to form a disease gene centralized protein–protein interaction network. Those genes with the largest confidence score (= 1) and the highest relevance score (= 1) to the OI pathogenic genes were selected to be interacting genes for OI. We then constructed a sub-network of pathogenic proteins and their interacting protein partners and focused on those interacting proteins (the predicted gene set A) that directly interact with more than one OI pathogenic proteins. In addition, we used WebGestalt to conduct pathway enrichment analysis based on Wikipathway database and selected those significant pathways (*P* < 0.05). Subsequently, we mapped those interacting gene partners of OI pathogenic genes into those significant pathways and selected those gene partners (the predicted gene set B) involved in these pathways. We then checked the expression patterns of those predicted OI-related pathogenic genes (the union of predicted gene set A and predicted gene set B). Last, we conducted literature survey to further verify those novel candidate risk genes. The whole process was displayed in **Figure 1B**.

### Statistical Analysis

Two-sided Fisher’s exact test was used to test the difference (*P* < 0.05) between the Gly-substitution missense and other types of variation (frameshift, nonsense, splicing) among OI subtypes, gender, pathogenic genes, and the presence of each clinical features in OI patients. One-sided Fisher’s exact test was used to test co-occurrence relationship (*P* < 0.05) between two different phenotypes. All the calculated progress was conducted by R software 3.4.3.

## Result

We extracted all experimentally confirmed OI-related pathogenic genes from the OIVD, and those pathogenic genes are *COL1A1*, *COL1A2*, *BMP1*, *CRTAP*, *P3H1*, *PPIB*, *TMEM38B*, *SERPINH1*, *FKBP10*, *PLOD2*, *IFITM5*, *SERPINF1*, *WNT1*, *CREB3L1*, *SP7*, *SPARC*, *MBTPS2*, *P4HB*, *PLS3,* and *SEC24D*. As less reported cases available for other 18 pathogenic non-collagen genes, we just collected cases with the variations reported in collagen gene *COL1A1* and *COL1A2* from literature.

Currently, there are four different subtypes (OI type I to IV) mainly caused by *COL1A1* and *COL1A2*, which correlate with different defects on type I collagen caused by different variation types (Forlino and Marini, 2016). We classified all variations into two variation categories (Rauch et al., 2010; Lin et al., 2015; Lindahl et al., 2015). The first variation type is missense in *COL1A1*/*COL1A2*, which mainly includes the Gly-substitution missense in the triple helix, leading to qualitative effects on type I collagen. The second variation type includes frameshift, nonsense, and splicing variations in *COL1A1*/*COL1A2* (Lin et al., 2015), causing a quantitative influence on type I collagen. The type of every variation in all patients was determined based on the results from literature and OIVD. Variations without recorded variation type were excluded. Among all the 155 patients, there were 59 Gly-substitution missenses, 70 variations of frameshift, nonsense, and splicing, and 26 non–Gly-substitution missenses or without variation type. We just analyzed 129 patients with Gly-substitution missense, frameshift, nonsense, or splicing variation.

### Genotype–Phenotype Association Analysis

To explore the associations between genotypes of pathogenic genes and OI clinical phenotypes, we first standardized the phenotypic description based on our eRAM system for those collected 155 patients (**Supplementary Table 1**), such as bone deformity, dense metaphyseal bands, blue sclera, hearing loss, and so on, and obtained 12 phenotypic categories. Among 129 analyzed patients, there were 49 OI type I patients, 1 OI type II patient, 10 OI type III patients, 29 OI IV patients, and 40 patients without defined OI type. The OI type II is generally perinatal lethal type, and the data for this type were insufficient and excluded for subsequent analysis.

Based on the two types of variation, we performed statistical significance test between the two variation types and 12 phenotypes recorded in the collected 129 cases. As a result, these two variation groups were significantly different among OI type I, OI type III, and OI type IV (*p* = 1.526e-07), most OI type I were caused by the second variation type (frameshift, nonsense, and splicing). The second variation type seemed to occur mainly in *COL1A1* (*p* = 1.833e-13), and no variation type difference was observed between male and female patients (*p* = 0.7218).

Among those 12 phenotypes, blue sclera was one of the most common phenotypes in OI disease. The number of patients with blue sclera was up to 128, accounting for 99.2% of 129 patients. However, there were no significant differences between two variation types in blue sclera (*p* = 0.4243) (**Table 1A**). In addition, no significant difference was observed between patients with the Gly-substitution missense and patients with other variations (frameshift, nonsense and splicing) for hypermobile joints (*p* = 1), dense metaphyseal bands (*p* = 0.4231), vertebral fracture (*p* = 1), osteopenia (*p* = 1), hearing loss (*p* = 0.6024), triangular face (*p* = 0.389), and popcorn calcif (*p* = 1) (**Table 1A**).

**Table 1:** Statistical analysis result on genotype–phenotype correlation and phenotype–phenotype correlation.

(A) Relationship between clinical characteristics and different variation types (Gly-substitution mutation vs. Frameshift, nonsense, and splicing mutation) in *COL1A1* and *COL1A2* of 129 patients with OI.				
Phenotype	Gly-substitution missense (n = 59)	Other variation (Frameshift, nonsense, and splicing) (n = 70)	Total case (Gly/other)	*P* value
**OI Type(I/III/IV)**	23.7%/23.7%/52.6%	80%/2%/18%	38/50	**1.53E-07**
Gender(F/M)	54.2%/45.8%	58.6%/41.4%	59/70	0.7218
***COL1A1*** **/** ***COL1A2* mutation**	40.7%/59.3%	97.1%/2.9%	58/70	**1.83E-13**
**Bone deformity**	80%	58.50%	45/53	**0.02946**
Hypermobile joints	83.3%	100%	12/3	1
Dense metaphyseal bands	81.8%	50.0%	11/2	0.4231
Vertebral anomalies	51.1%	31.3%	45/48	0.06021
Vertebral fracture	40.0%	42.1%	20/19	1
Osteopenia	100%	100%	1/2	1
**Dentinogenesis imperfecta**	56.9%	35.2%	51/54	**0.03189**
Blue sclera	70.7%	77.1%	58/72	0.4243
Hearing loss	14.0%	18.5%	50/54	0.6024
**Walking with assistance**	31.0%	0%	29/42	**0.0001345**
Triangular face	25.8%	16.7%	31/42	0.3889
Popcorn calcif	25.0%	0%	12/2	1
(B) Relationship between dentinogenesis imperfecta and bone deformity/vertebral anomalies. Phenotype with significant difference (*p* < 0.05) are represented in bold font.				
Phenotype	Dentinogenesis imperfecta (Y)	Dentinogenesis imperfecta (N)	Total case (DI(Y)/DI(N))	*P* value
**Bone deformity**	85.10%	52.90%	47/51	**0.0005576**
**Vertebral anomalies**	53.50%	30%	43/50	**0.01832**

Results revealed that patients with the Gly-substitution missense tended to develop bone deformity (*p* = 0.02946) and DI (*p* = 0.03189). In addition, we also found that patients with DI were prone to have bone deformity (*p* = 0.0005576) or vertebral anomalies (*p* = 0.01832) (**Table 1B**). Scoliosis, which composes part of vertebral anomalies, is one of the mostly common phenotypes in OI. Previous study also indicated that children with DI had a large probability in having scoliosis, pathological kyphosis, and basilar impression (Engelbert et al., 1998). The association between the first mutation type and bone deformity/DI provides new insight into the research on spinal-related abnormalities disease, like spinal complications. Although DI had a strong relationship with vertebral anomalies, no significant difference was observed between two variation types in vertebral anomalies (vertebral anomalies, *p* = 0.06021). In addition, patients with the Gly-substitution missense had a poorer walking ability (*p* = 0.0001345).

### Novel Candidate Pathogenic Variations Identification

**Figure 2:**
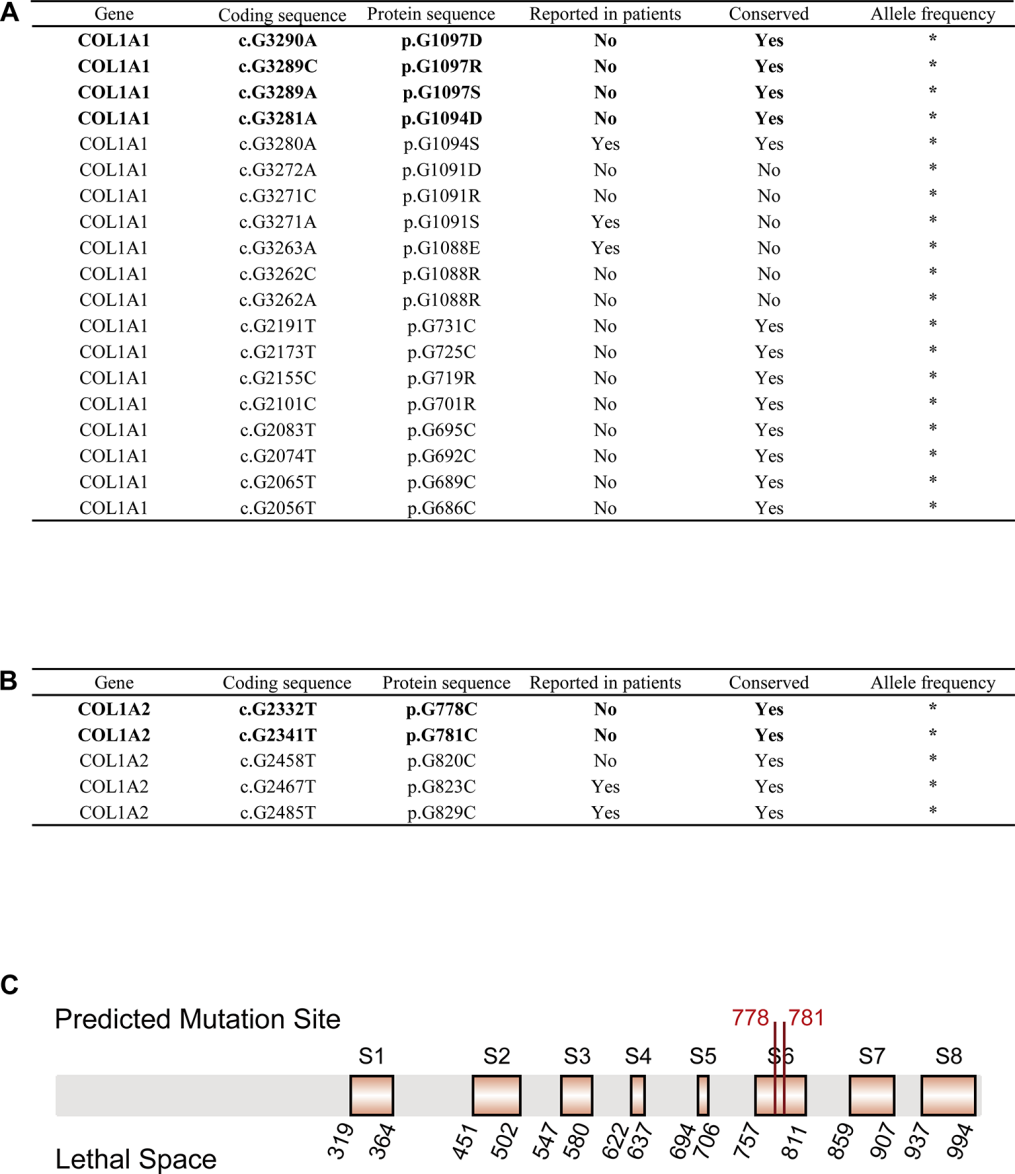
OI-related candidate mutations in *COL1A1* and *COL1A2*. **(A)** OI-related candidate mutations in *COL1A1* (“*”: currently unknown). **(B)** OI-related candidate mutations in *COL1A2* (“*”: currently unknown). **(C)** Localization of variants c.G2332T (p.G778C) and c.G2341T (p.G781C) in *COL1A2* protein sequence. Predicted pathogenic variations are represented in bold font (Eight orange rectangles with black border denote eight lethal spaces).

We obtained 55,032 possible mutations of *COL1A1* and 110,016 possible mutations of *COL1A2* in ANNOVAR. After filtering out mutations based on the cutoff value (SIFT_score = 0, Polyphen2_HVAR_score = 1, CADD_phred > 30, GERP++_RS > 5), 19 *COL1A1* mutations and 5 *COL1A2* mutations were kept as pathogenic variations. Strikingly, 5 of the 24 mutations have been reported to be pathogenic to OI in ClinVar, dbSNP or OIVD Database (**Figures 2A, B**). The remaining 16 candidate mutations in *COL1A1* and three candidate mutations in *COL1A2* currently have no supporting evidence.

Next, we checked the frequency of those candidate variations in gnomAD and CMBD databases, and no frequency has been reported in any ethnic groups. The result indicated that all the variations are an extremely rare site with high probability of lethality for the disease. The multiple sequence alignment of *COL1A1*/*COL1A2* homologous genes among seven species (*Homo sapiens*, *Pan troglodytes*, *Papio anubis*, *Macaca mulatta*, *Mus musculus*, *Xenopus laevis*, *Caenorhabditis elegans*) with COMBALT revealed that 12 mutations in *COL1A1* and 3 mutations in *COL1A2* located in highly conserved regions among those species. Finally, we mapped the 15 candidate variations into the domains of α1(I) chain/α2(I) chain (encoded by *COL1A1*/*COL1A2*) in Ensembl, and found only four mutations (c.G3290A (p.G1097D), c.G3289C (p.G1097R), c.G3289A (p.G1097S), c.G3281A (p.G1094D)) in *COL1A1* located in collagen triple helix repeat domain, which influence type I collagen formation. In contrast, two mutations (c.G2332T (p.G778C), c.G2341T (p.G781C)) in *COL1A2* were not in any domain region, but located in two reported lethal spaces in *COL1A2* protein sequence. We supposed that these two candidate variations also associate with OI disease.

### Novel Candidate Risk Genes Identification

**Figure 3:**
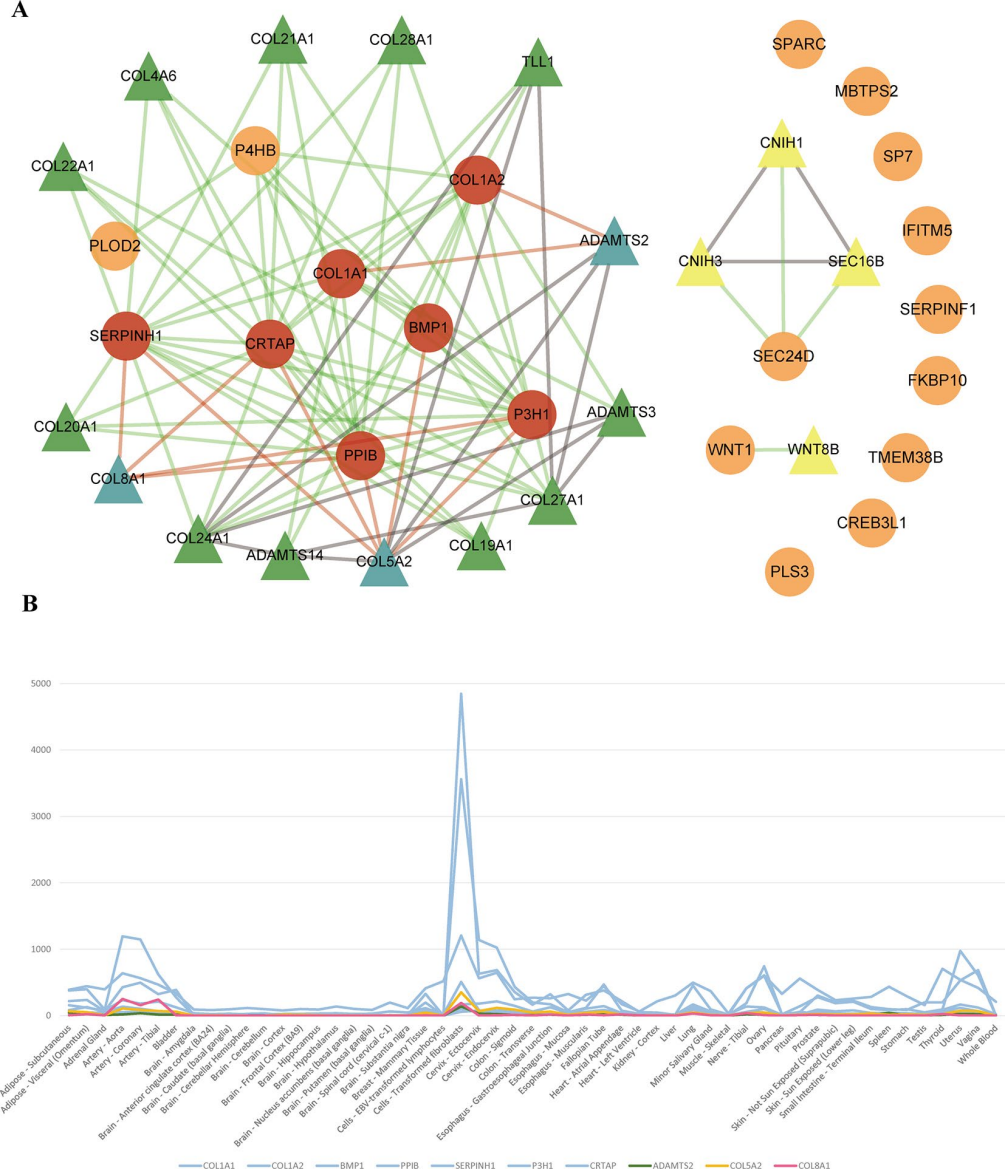
**(A)** Network of OI pathogenic genes and candidate genes. 93 interactions between 20 OI pathogenic genes and 18 interacted genes. The circles and triangles represent 20 OI pathogenic genes and 18 interacted genes (≥1 interaction), respectively. Yellow triangles denote four predicted genes (= 1 interaction). Green and blue triangles totally denote 14 predicted genes (the predicted gene set A) (≥2 interactions), and blue triangles denote three candidate genes (gene expression is consistent with OI pathogenic genes), respectively. Red circles denote seven OI pathogenic genes (interact with three candidate genes). Grey lines denote 15 interactions between predicted genes. Green lines and red lines totally denote 78 interactions between OI pathogenic genes and predicted genes, and red lines denote 11 interactions between three candidate genes and seven interacted OI pathogenic genes, respectively. **(B)** The tissue expression distribution of predicted genes and their interacted pathogenic genes. The green, yellow, red, and blue lines denote candidate gene *ADAMTS2*, *COL5A2*, *COL8A1* and interacted pathogenic genes (*COL1A1*, *COL1A2*, *BMP1*, *PPIB*, *SERPINH1*, *P3H1*, *CRTAP*). All these genes are expressed highly in “Cells_Transformed_fibroblasts” and have a similar expression distribution among all human tissues.

To identify new risk genes related to OI, we carried out network-based integrative analysis, the assumption of our approach is that if a protein directly interacts with more than one OI causative proteins or is enriched in the same pathway with known causal genes, theoretically, the protein would be a candidate that also contributes to OI disease. Therefore, we first mapped all 20 pathogenic genes into the InWeb_InBioMap database to construct a PPI network, then we selected those proteins with confidence score cutoff (= 1) and relevance score cutoff (= 1). The resulting network contains 20 pathogenic proteins with 18 directly interactive partners (*ADAMTS2, ADAMTS3, ADAMTS14, COL4A6 COL5A2, COL8A1, COL19A1, COL20A1, COL21A1, COL22A1, COL24A1, COL27A1, COL28A1, TLL1, CNIH1, CNIH3, SEC16B, WNT8B*) (≥1 interaction) (**Figure 3A**). We extracted 14 interactive proteins (*ADAMTS2, ADAMTS3, ADAMTS14, COL4A6, COL5A2, COL8A1, COL19A1, COL20A1, COL21A1, COL22A1, COL24A1, COL27A1, COL28A1, TLL1*) that interact with more than one pathogenic proteins from PPI network as the predicted gene set A (≥2 interactions). Next, we performed pathway enrichment analysis on 20 disease-causing genes and obtained 18 significant pathways (*p* < 0.05). Among the 18 interacting gene partners, 3 of them (*COL4A6, COL5A2, WNT8B*) fell into those 18 significant pathways (the predicted gene set B) (**Table 2**). Based on the integrated result of the protein–protein network (14 interactive proteins from the predicted gene set A) and pathway enrichment analysis (3 interactive proteins from the predicted gene set B), we finally identified 15 genes (*ADAMTS2, ADAMTS3, ADAMTS14, COL4A6, COL5A2, COL8A1, COL19A1, COL20A1, COL21A1, COL22A1, COL24A1, COL27A1, COL28A1, TLL1, WNT8B*) as the potential causal genes to OI disease. To further verify those predicted genes for OI disease, we checked the expression pattern of those 15 genes in different human tissues based on GTEx data. As related to collagen hereditary disease, most of the OI causative genes are expressed in artery aorta and transformed fibroblasts (**Supplementary Figure**). Artery aorta is rich in connective tissue, and fibroblasts secrete a variety of substrates, collagen and fibers. After filtering out those predicted genes that do not show similar tissue expression pattern with that of most pathogenic genes, only three candidate genes were left (*ADAMTS2*, *COL5A*2, and *COL8A1*). Those three candidate genes presented similarly tissue expression pattern with their seven interactive OI pathogenic genes (*COL1A1*, *COL1A2*, *PPIB*, *SERPINH1*, *P3H1*, *BMP1*, *CRTAP*), especially showed high expression in transformed fibroblasts (**Figure 3B**).

**Table 2:** Significant pathways with related pathogenic genes and predicted genes.

Significant Pathways	20 Pathogenic Genes and 18 Interacted Genes	*P* value
Epithelial to mesenchymal transition in colorectal cancer	**COL4A6**, **WNT8B**, SPARC, WNT1	0.00083567
Focal Adhesion-PI3K-Akt-mTOR-signaling pathway	**COL4A6, COL5A2**, COL1A1, COL1A2, CREB3L1	0.0010862
miR-509-3p alteration of YAP1/ECM axis	COL1A1, SPARC	0.0011611
Focal Adhesion	**COL4A6, COL5A2**, COL1A1, COL1A2	0.0018166
Inflammatory Response Pathway	COL1A1, COL1A2	0.0035058
miRNA targets in ECM and membrane receptors	**COL5A2**, COL1A2	0.0059006
PI3K-Akt Signaling Pathway	**COL4A6**, COL1A1, COL1A2, CREB3L1	0.012283
Sterol Regulatory Element-Binding Proteins (SREBP) signaling	MBTPS2, SEC24D	0.01637
Hypothetical Craniofacial Development Pathway	WNT1	0.021586
ncRNAs involved in Wnt signaling in hepatocellular carcinoma	SERPINF1, WNT1	0.023282
LncRNA involvement in canonical Wnt signaling and colorectal cancer	SERPINF1, WNT1	0.031757
Senescence and Autophagy in Cancer	COL1A1, SPARC	0.034042
EDA Signaling in Hair Follicle Development	BMP1	0.037488
Osteoblast Signaling	COL1A1	0.037488
Wnt Signaling Pathway	SERPINF1, WNT1	0.040029
Canonical and Non-Canonical TGF-B signaling	BMP1	0.045347
Regulation of Wnt/B-catenin Signaling by Small Molecule Compounds	WNT1	0.047953
Adipogenesis	BMP1, WNT1	0.04837

Eighteen significant pathways (P < 0.05) were identified with 20 OI causative genes and their 18 interacting gene partners from protein–protein interaction network. Candidate genes (the predicted gene set B) are represented in bold font.

## Discussion

OI is a rare disease with bone disorders characterized by bone fragility and skeletal deformity. Clinical observation indicates that currently identified pathogenic genes and variations cannot fully decipher the phenotypic and genetic heterogeneity of the disease. To better explore the underlying mechanism of OI, we performed genotype and phenotype association analysis based on manually collected 155 patients’ data. According to the variation type, we classified mutations into two groups, one group refers to the Gly-substitution missense, and another includes frameshift, nonsense, and splicing mutation. Most of the Gly-substitution missenses result in structural abnormalities of type I collagen, whereas the second mutation group caused by the early termination of codons lead to insufficient collagen synthesis. Our results showed that the two mutation types played different roles in bone deformity, DI, and walking with assistance, which was consistent with the results of other studies (Lin et al., 2015).

In addition, we found that patients with DI were more likely to have bone deformity (*p* = 0.0005576) and vertebral anomalies (*p* = 0.01832). Similarly, Lin et al. also reported that patients with DI were more susceptible to bone deformities and scoliosis (Lin et al., 2015). Most of the patients in our collected data with both two phenotypes contain the Gly-substitution missense (DI and bone deformity: 62.5%; DI and vertebral anomalies: 78.3%). Based on the observation, we supposed that the collagen structural abnormalities caused by the Gly-substitution missense could result in the abnormal development of the whole body bone morphology, which could also be one of the pathogenic factors for other bone disorders. Especially, the strong co-occurrence of DI and vertebral anomalies implied that *COL1A1* and *COL1A2* genes could also contribute to other spinal and vertebral diseases.

For the 24 pathogenic mutations from ANNOVAR annotation, we searched different variation annotation databases, including ClinVar, dbSNP, and OIVD, to explore whether they have any disease-related report. As a result, 19 of the mutations have no report linked to OI patients. However, multiple alignment with homologous genes from different species with COMBALT showed that only six variations (*COL1A1*: c.G3290A (p.G1097D), c.G3289C (p.G1097R), c.G3289A (p.G1097S), and c.G3281A (p.G1094D); *COL1A2*: c.G2332T (p.G778C) and c.G2341T (p.G781C)) locate in the conserved regions, which indicates that these four positions (amino acid position: 1094 and 1097 (on α1(I) chain); 778 and 781 (on α2(I) chain)) have a significant impact on protein structure or function. Among the 6 candidate variations, four of them (in COL1A1) locate in the triple helical region and the other two variations (in COL1A2) locate in the collagen region.

**Figure 4:**
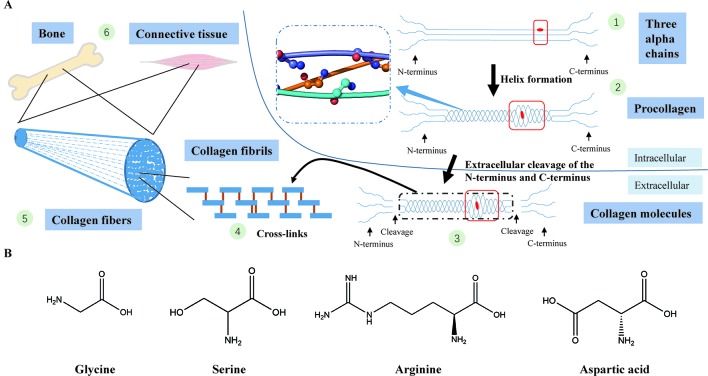
**(A)** Type I collagen synthesis and application. (1) Formation of three alpha chains (two α1(I) chains and one α2(I) chain) (the red dot represents a Gly-substitution missense). (2) Triple helix formation (the blue dashed-line box displays the structure of the triple helix with a smooth loop model, in which all glycine residues are shown in ball-and-stick representation; the red box indicates abnormality of the triple helix after the Gly replacement). (3) Extracellular cleavage of the N-terminus and C-terminus (the red box indicates abnormality of the triple helix after the Gly replacement). (4) Cross-linking of type I collagen molecules. (5) Assembly of collagen fibrils to collagen fibers. (6) Collagen fibers participate in the formation of bone and connective tissues. **(B)** The chemical structural formula of four amino acids (glycine, serine, arginine, asparagic acid). Glycine, which has the smallest relative molecular mass, is the only amino acid with no sidechain.

A collagen triple helix is formed by three chains (two α1(I) chains and one α2(I) chain) supercoiling around the common axis and glycine, framing almost 338 Gly-Xaa-Yaa repeats in the region, is the only residue small enough to be accommodated in the limited interior of the helical space (Ramachandran and Kartha, 1955; Rich and Crick, 1961; Brodsky and Persikov, 2005). In the collagen triple helix, the Gly-substitution missense will produce structural deformation of the triple helix, leading to destabilization of the helical structure, affecting the synthesis of collagen (**Figure 4**) (Brodsky and Persikov, 2005; Qiu et al., 2018).

To validate the pathogenicity of the candidate variations in *COL1A1*, we checked the specificity of their locations (positions of the four candidate mutations: 1094 and 1097). Evidence from the protein families database (Pfam) (El-Gebali et al., 2019) demonstrate that the locations of all four variations belong to the collagen triple helix region (PF01391: Collagen triple helix repeat (1079–1137)). Structurally, different abnormalities in the collagen helix are associated with the identity of the residue replacing Gly (Bryan et al., 2011; Qiu et al., 2018), which also influence the severity of OI patients (residues replacing Gly of four candidate mutations: Asp, Arg, and Ser). Through the statistical analysis on the location of Glysubstitution mutations in a large number of OI patients, Beck et al. found that all Gly→Asp in the α1(l) chain led to OI type II (perinatal lethat form) (Beck et al., 2000). In addition, the study of the impact of various Gly replacements discovered that the three replaced form (Gly→Arg, Gly→Ser, and Gly→Cys) had a stronger association with OI lethality than the other replaced forms (Beck et al., 2000). In all, these conclusions indicate that the four candidate mutations of *COL1A1* we identified are highly likely to cause lethal OI phenotypes.

For the two candidate variations in *COL1A2*, we found that the two mutations locate in a special region which is enriched with lethal mutations. Previous study reported that OI-related lethal mutations normally accumulate in eight regularly spaced clusters along the chain α2(I) (Marini et al., 2007). Truly, both c.G2332T (p.G778C) and c.G2341T (p.G781C) locate in the lethal space 6 (S6) (**Figure 2C**) which belongs to the binding region of proteoglycans (keratan sulfate and heparan sulfate proteoglycan) and type I collagen fibril, the deformation of this region affects the interaction between type I collagen and other components in extracellular matrix (San Antonio et al., 1994; Schaefer, 2014). Abnormalities in this region can lead to abnormal binding of collagen to the matrix, which will affect some of the corresponding biological functions, such as the signal interruption or the distortion of the three-dimensional framework of tissue and organ molecules (Bella and Hulmes, 2017). These biological function disorders then affect bone development and differentiation, leading to bone abnormalities (Schaefer, 2014). Therefore, we conclude that these two variations possibly generate a severe effect on people carrying the mutations, and are most likely related to OI. In summary, those evidences all support the pathogenesis of these six candidate mutations to OI.

Among the three identified potential new risk factors to OI, *ADAMTS2* and *COL5A2* seem to be more associated with OI than *COL8A1*. In fact, *ADAMTS2* is highly expressed in the skin, bones, tendons, and aorta and has a strong correlation with type I collagen (Bekhouche and Colige, 2015). When procollagen is transferred to the extracellular space, the amino-terminal-propeptide (N-terminal propeptide) and carboxy-terminal propeptide (C-terminal propeptide) at both ends of procollagen would be proteolytically removed to form collagen fibrils (Nijhuis et al., 2019). Next, a large number of collagen fibrils are aggregated into collagen fibers, and finally assembled into the structural framework of cells and tissues and bone (**Figure 4A**) (Kadler et al., 1996; Van Dijk and Sillence, 2014; Bekhouche and Colige, 2015; Marini et al., 2017). *ADAMTS2* encodes procollagen I N-proteinase, which is used to excise the N-terminal propeptides in procollagen. Abnormal expression of the *ADAMTS2* gene will lead to accumulation of pN-procollagen (collagen molecules with an N-terminal propeptide sequence that has not been cleaved), which will result in the polymerization of the abnormal collage fibers (Nusgens et al., 1992; Colige et al., 1997; De Coster et al., 2007). According to the phenotypic level, it has been reported that mutations in *ADAMTS2* caused type I collagen disorders, resulting in DI (one feature of OI) (De Coster et al., 2007). Evidences from Mouse Genome Informatics (MGI) database (Eppig, 2017; Law and Shaw, 2018) also demonstrate that *ADAMTS2* mutated mice show thin skin, triangular face, abnormal cutaneous collagen fibril morphology, abnormal hair follicle morphology, and other phenotypes. Similarly, we noticed that two phenotypes “thin skin” and “triangular face” also exist in the human phenotypes of OI, which further supports the conclusion that *ADAMTS2* gene is most likely to be associated with OI (Marini et al., 2017).

*COL5A2*, which encodes type V collagen, plays an important role in tissue-specific matrix assembly and the regulation of fibrillogenesis (Wenstrup et al., 2004). From a molecular point of view, most collagen fibril consist of a large number of type I collagen and a very small number (∼2%) of type V collagen. Although type V collagen only occupies small portion of collagens in all tissues, it is crucial for collagen fibril nucleation, regarded as collagen fibrillogenesis (Connizzo et al., 2015; Makuszewska et al., 2019). Mouse model results indicate that the deficiency or abnormality of type V collagen leads to a lack of fibril formation in mouse embryonic tissue and even death in the stage of early embryos (Wenstrup et al., 2004; Connizzo et al., 2015), which is similar to the phenotypes in human OI type II (perinatal lethal type). Evidences in MGI shows that mice with *COL5A2* mutation present abnormal cardiovascular system physiology, abnormal skeleton development, abnormal cutaneous collagen fibril morphology, abnormal cornea morphology, embryonic lethality during organogenesis, neonatal lethality, respiratory distress, thin dermal layer, and other phenotypes (Chen et al., 2017), most of these phenotypes are very similar to the human phenotypes of OI, therefore, we suggest that the *COL5A2* gene is tightly correlated with OI, especially OI type II.

Type VIII collagen, co-encoded by *COL8A1* and *COL8A2*, is a major component of the Descemet’s membrane of corneal endothelial cells and is also expressed in other tissues, such as the cornea, sclera, blood vessels, heart, kidneys, and lungs (Hopfer et al., 2005). One clinical feature of OI is the blue sclera, OI also presents thin corneal thickness, smaller corneal diameter, retinal detachment, corneal opacities, myopia, smaller globe length, primary open-angle glaucoma, and other eye abnormalities (Wallace et al., 2014; Hald et al., 2018; Lagrou et al., 2018). Dimasi et al. performed an eye measurement on 28 OI type I patients and found that their mean central corneal thickness (CCT) was lower than that of people in the normal population (Dimasi et al., 2010), indicating that there is a correlation between OI type I and low CCT. Previous studies revealed that mutations in type VIII collagen could lead to lower CCT and thinner Descemet’s membrane in the Caucasian and Asian populations (Desronvil et al., 2010). In addition, based on the evidence from MGI, *COL8A1* mutated mice show decreased cornea thickness, abnormal Descemet membrane and other eye abnormalities, we suppose that *COL8A1* is associated with low CCT. In conclusion, *COL8A1* might be associated with eye abnormalities and could also be related to OI.

Taken together, we have explored the association between genotypes and phenotypes in OI with the collected cases from literature. We also have systematically analyzed the impact of each predicted variants in pathogenic genes and identified the potential risk genes for OI, which provide new insights into the underlying mechanism of OI disease. However, our method also has certain limitations, one of them is that many cases do not have their clinical phenotypes fully recorded, which resulted in the insignificant association between certain genotypes and phenotypes. Meanwhile, the phenotypes described in different patients could also be inconsistent. In addition, ANNOVAR is only focused on coding region, which make the non-coding region unavailable for pathogenic analysis. Nevertheless, our research provides an alternative way to study a new mechanism for rare diseases (Jia and Shi, 2017). Our findings should shed light on the better understanding of OI disease and its effective disease diagnosis.

## Author Contributions

In this study, TS, YG, and XN designed the study. JS, MR, JJ and MT conducted the data collection and data analysis. JS, MR, JJ, TS, YG, and XN interpreted data in context of BSCL biology. JS, MR, and JJ drafted the manuscript. TS, XN, and YG revised and finalized the manuscript. All authors read and approved the final manuscript.

## Funding

This work was supported by the China Human Proteome Project (Grant No.2014DFB30010, 2014DFB30030), National Natural Science Foundation of China (31671377, 81472369, and 81502144), Clinical Application Research Funds of Capital Beijing (Z171100001017051), Beihang University & Capital Medical University Advanced Innovation Center for Big Data-Based Precision Medicine Plan (BHME- 201804) and Shanghai 111 Project (B14019).

## Conflict of Interest

The authors declare that the research was conducted in the absence of any commercial or financial relationships that could be construed as a potential conflict of interest.
