# Corrigendum: Genotype–Phenotype Association Analysis Reveals New Pathogenic Factors for Osteogenesis Imperfecta Disease

**DOI:** 10.3389/fphar.2019.01603

**Published:** 2020-02-05

**Authors:** Jingru Shi, Meng Ren, Jinmeng Jia, Muxue Tang, Yongli Guo, Xin Ni, Tieliu Shi

**Affiliations:** ^1^ Center for Bioinformatics and Computational Biology, and the Institute of Biomedical Sciences, School of Life Sciences, East China Normal University, Shanghai, China; ^2^ Big Data and Engineering Research Center, Beijing Key Laboratory for Pediatric Diseases of Otolaryngology, Head and Neck Surgery, MOE Key Laboratory of Major Diseases in Children, Beijing Children's Hospital, National Center for Children's Health, Beijing Pediatric Research Institute, Capital Medical University, Beijing, China; ^3^ Biobank for Clinical Data and Samples in Pediatrics, Beijing Children's Hospital, National Center for Children's Health, Beijing Pediatric Research Institute, Capital Medical University, Beijing, China; ^4^ Department of Otolaryngology, Head and Neck Surgery, Beijing Children's Hospital, National Center for Children's Health, Capital Medical University, Beijing, China

**Keywords:** osteogenesis imperfecta, genotype, phenotype, novel candidate pathogenic genes, novel candidate pathogenic variations

There is an error in the **Funding** statement. The correct number for “Beihang University & Capital Medical University Advanced Innovation Center for Big Data-Based Precision Medicine Plan**”** is “BHME- 201804.”

Additionally, in the original article, there was one error. In the **Discussion** section, the locations of the four candidate pathogenic variations in *COL1A1* were incorrectly mapped to the major ligand-binding region (MLBR3).

A correction has been made to the **Discussion**, **Paragraph five**:

“To validate the pathogenicity of the candidate variations in *COL1A1*, we checked the specificity of their locations (positions of the four candidate mutations: 1094 and 1097). Evidence from the protein families database (Pfam) (El-Gebali et al., 2019) demonstrate that the locations of all four variations belong to the collagen triple helix region (PF01391: Collagen triple helix repeat (1079–1137)). Structurally, different abnormalities in the collagen helix are associated with the identity of the residue replacing Gly (Bryan et al., 2011; Qiu et al., 2018), which also influence the severity of OI patients (residues replacing Gly of four candidate mutations: Asp, Arg, and Ser). Through the statistical analysis on the location of Glysubstitution mutations in a large number of OI patients, Beck et al. found that all Gly→Asp in the α1(l) chain led to OI type II (perinatal lethat form) (Beck et al., 2000). In addition, the study of the impact of various Gly replacements discovered that the three replaced form (Gly→Arg, Gly→Ser, and Gly→Cys) had a stronger association with OI lethality than the other replaced forms (Beck et al., 2000). In all, these conclusions indicate that the four candidate mutations of *COL1A1* we identified are highly likely to cause lethal OI phenotypes.”

Due to the error outlined above, the citations for “Di Lullo et al., 2002” and “Xiao et al., 2015” have been removed from the reference list, and “El-Gebali et al., 2019” has been cited instead.

The authors apologize for these errors and state that these do not change the scientific conclusions of the article in any way. The original article has been updated.

## References

[B1] Beck K. Chan V. C. Shenoy N. Kirkpatrick A. Ramshaw J. A. Brodsky B. (2000). Destabilization of osteogenesis imperfecta collagen-like model peptides correlates with the identity of the residue replacing glycine. Proc. Natl. Acad. Sci. U. S. A. 97 (8), 4273–4278. 10.1073/pnas.070050097 10725403 PMC18226

[B2] Bryan M. A. Cheng H. Brodsky B. (2011). Sequence environment of mutation affects stability and folding in collagen model peptides of osteogenesis imperfecta. Biopolymers 96 (1), 4–13. 10.1002/bip.21432 20235194 PMC2980582

[B3] Di Lullo G. A. Sweeney S. M. Korkko J. Ala-Kokko L. San Antonio J. D. (2002). Mapping the ligand-binding sites and disease-associated mutations on the most abundant protein in the human, type I collagen. J. Biol. Chem. 277 (6), 4223–4231. 10.1074/jbc.M110709200 11704682

[B4] El-Gebali S. Mistry J. Bateman A. Eddy S. R. Luciani A. Potter S. C. (2019). The Pfam protein families database in 2019. Nucleic Acids Res. 47 (D1), D427–D432. 10.1093/nar/gky995 30357350 PMC6324024

[B5] Qiu Y. Mekkat A. Yu H. Yigit S. Hamaia S. Farndale R. W. (2018). Collagen Gly missense mutations: effect of residue identity on collagen structure and integrin binding. J. Struct. Biol. 203 (3), 255–262. 10.1016/j.jsb.2018.05.003 29758270 PMC6089640

[B6] Xiao J. Yang Z. Sun X. Addabbo R. Baum J. (2015). Local amino acid sequence patterns dominate the heterogeneous phenotype for the collagen connective tissue disease osteogenesis imperfecta resulting from Gly mutations. J. Struct. Biol. 192 (1), 127–137. 10.1016/j.jsb.2015.05.002 25980613 PMC4898063

