# Supplementary material for: Genotype–Phenotype Association Analysis Reveals New Pathogenic Factors for Osteogenesis Imperfecta Disease
Source: Front Pharmacol. 2019 Oct 15;10:1200. doi: 10.3389/fphar.2019.01200 (PMC6803541; doi:10.3389/fphar.2019.01200)

Supplementary Figure: Tissue expression distribution of 20 OI pathogenic genes and 15 predicted genes.

[20 OI pathogenic genes]:

1. COL1A1

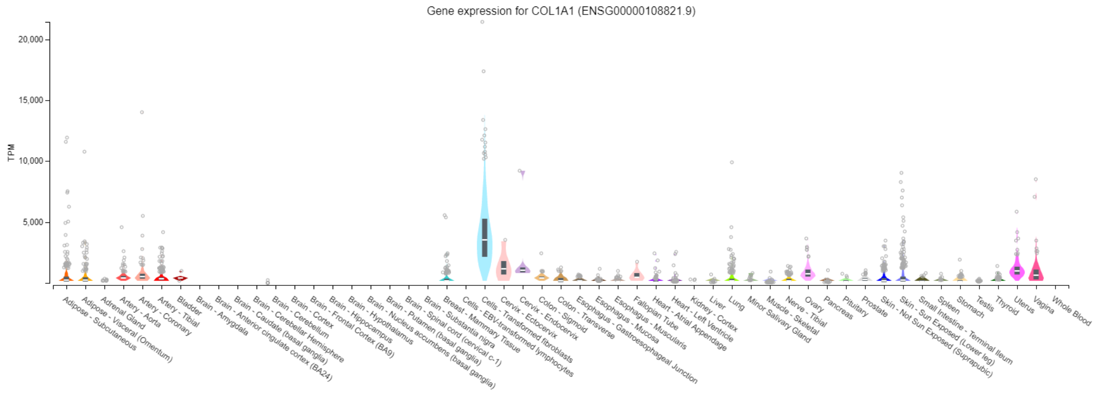

2. COL1A2

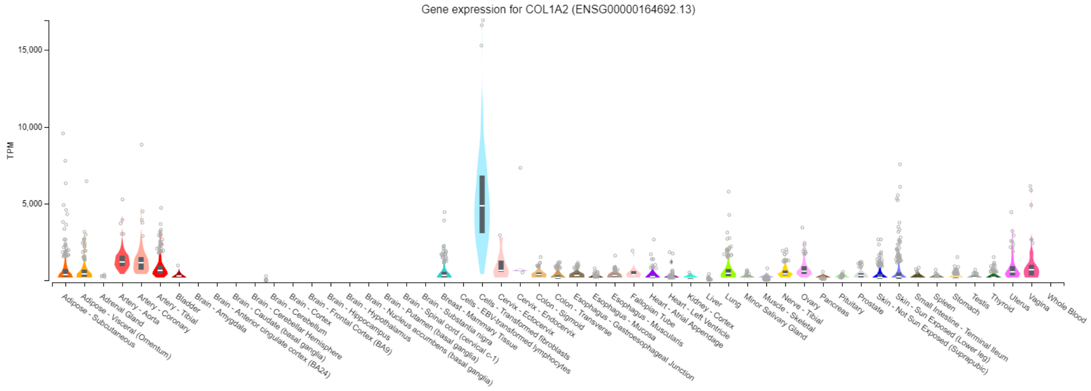

3. BMP1

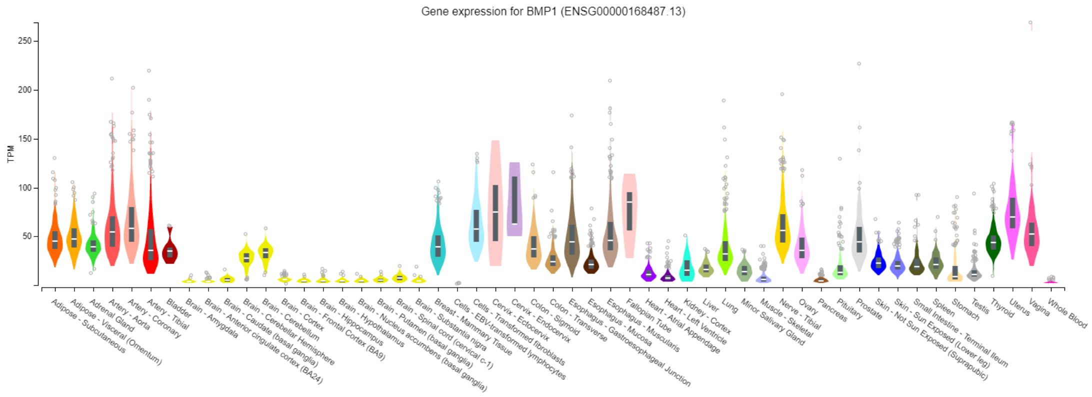

4. CRTAP

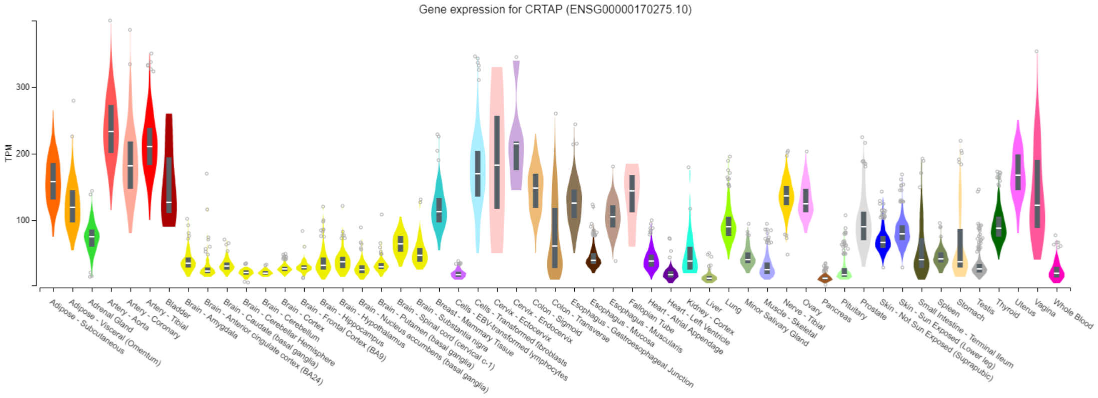

5. P3H1 (LEPRE1)

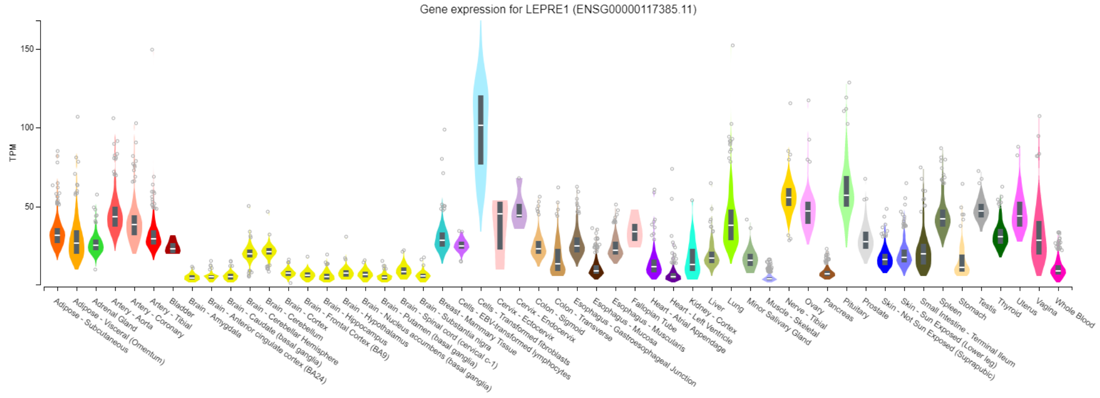

6. PPIB

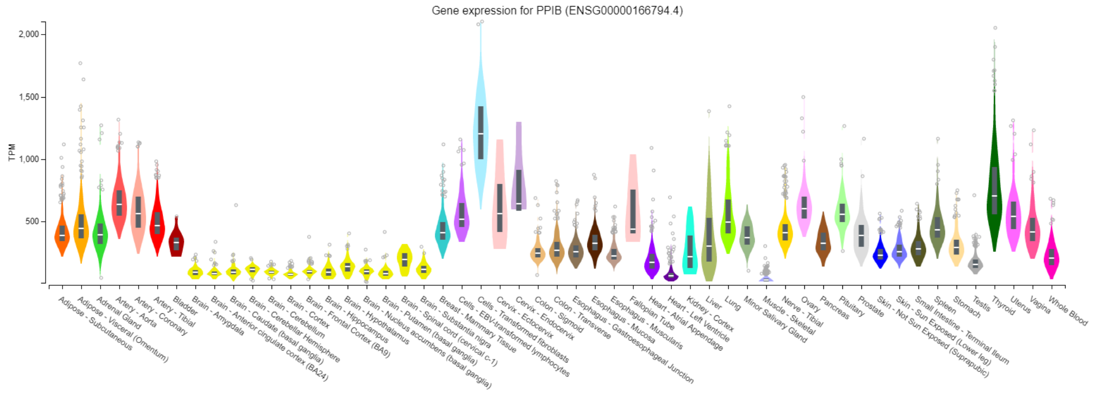

7. TMEM38B

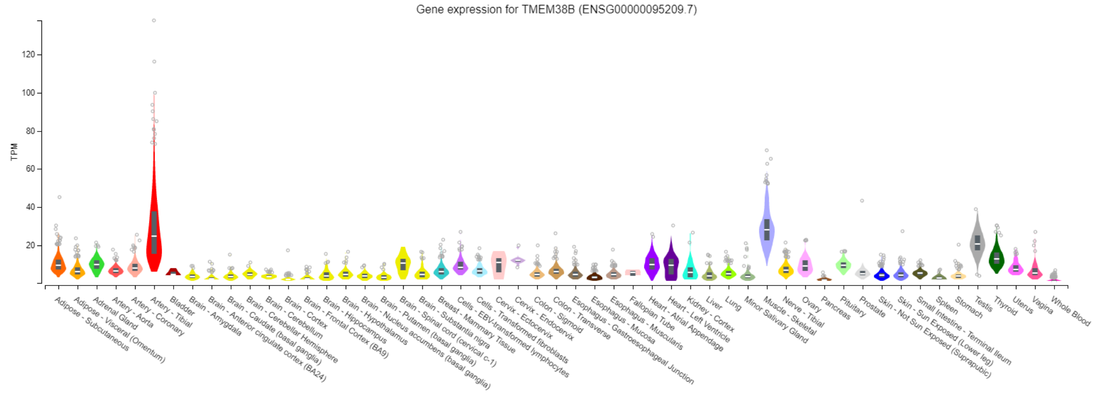

8. SERPINH1

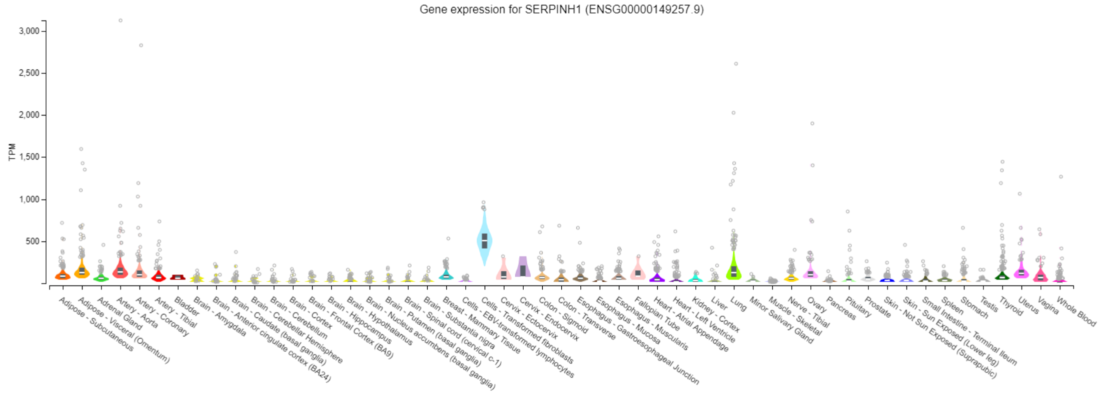

9. FKBP10

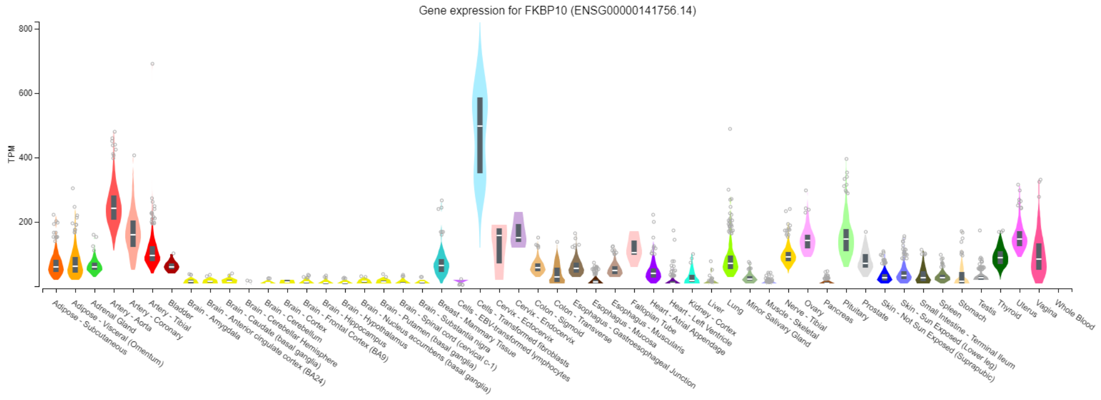

10. PLOD2

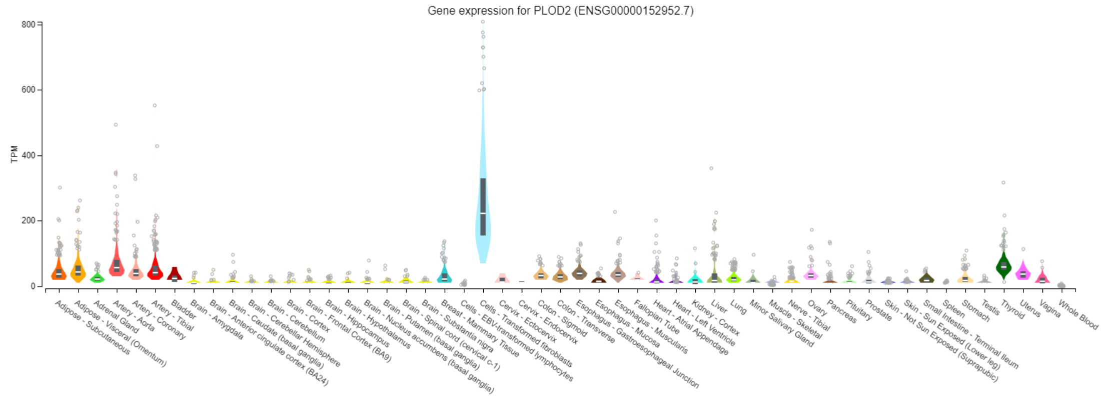

11. IFITM5

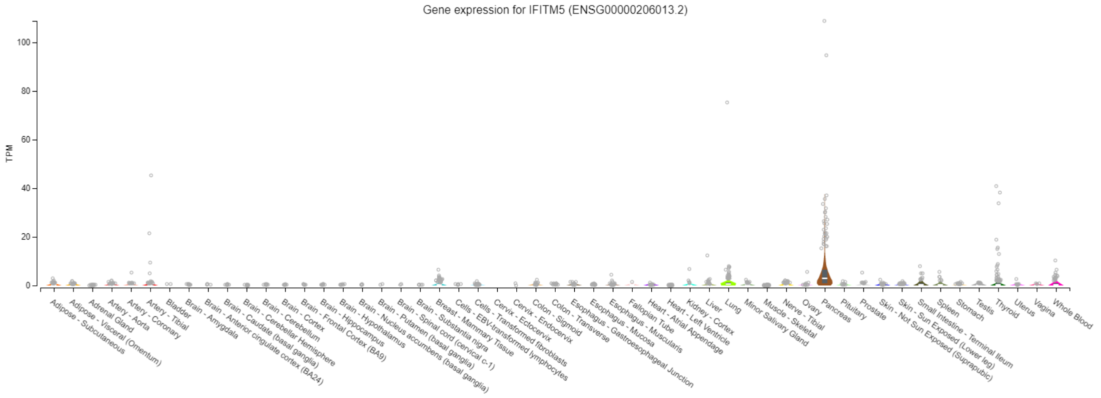

12. SERPINF1

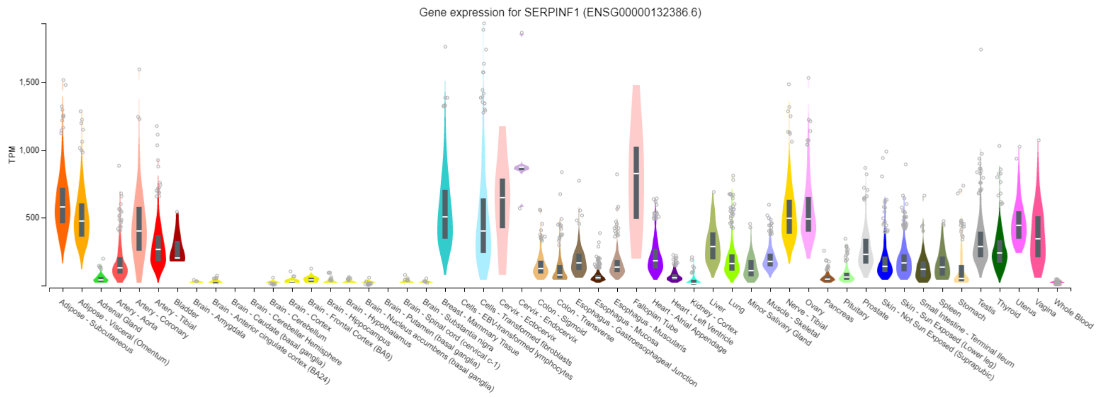

13. WNT1

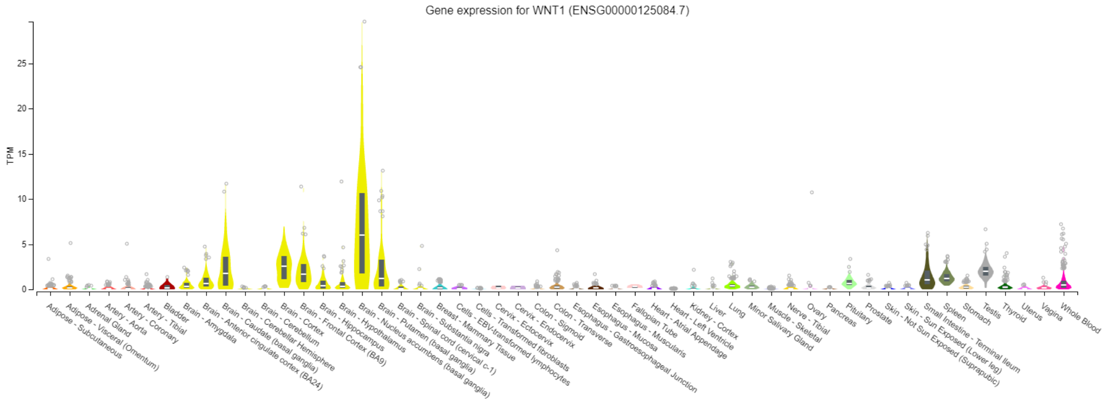

14. CREB3L1

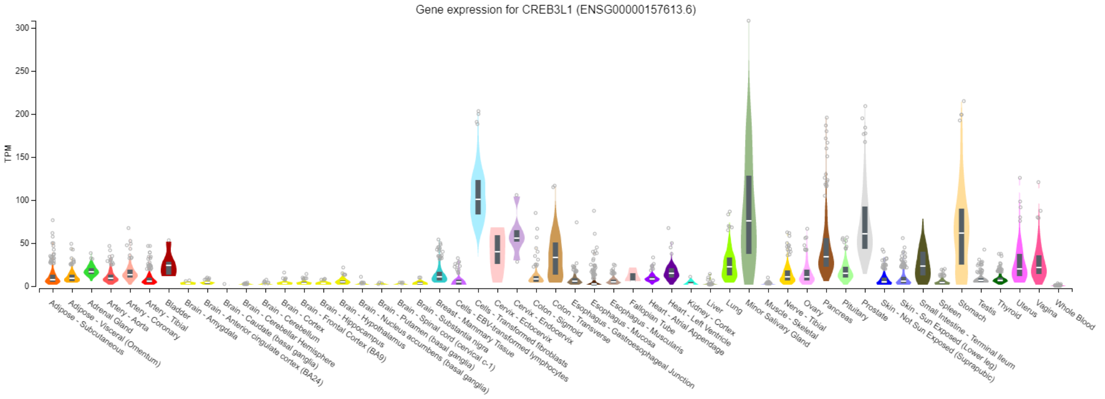

15. SP7

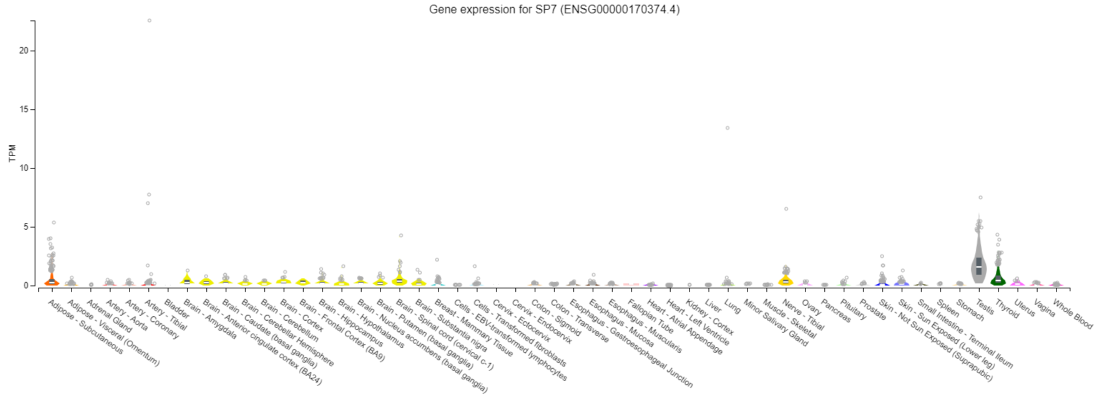

16. SPARC

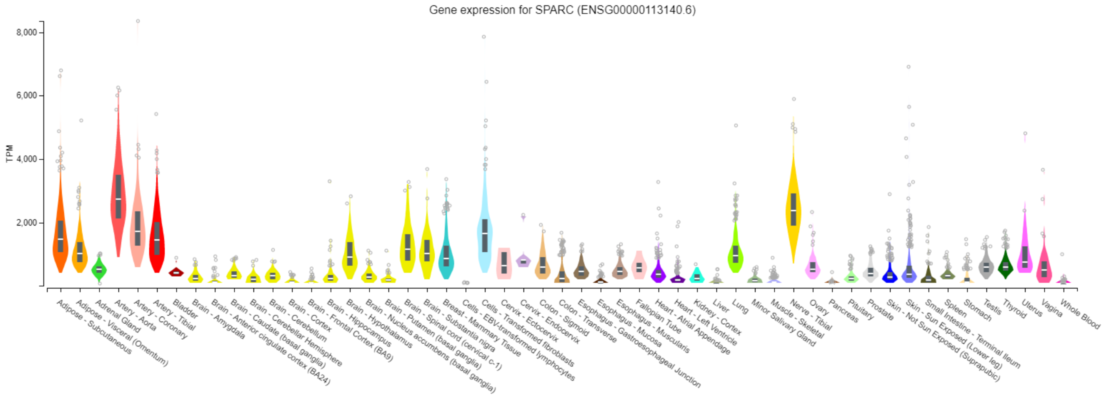

17. MBTPS2

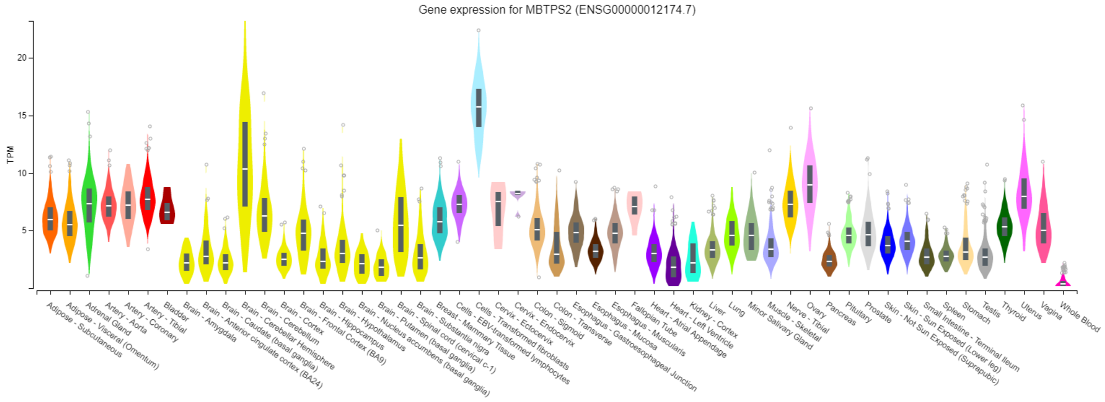

18. P4HB

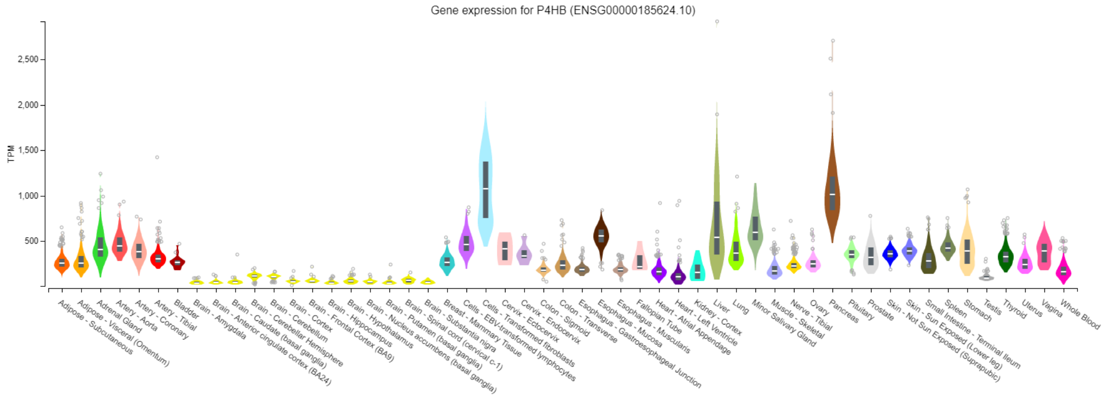

19. PLS3

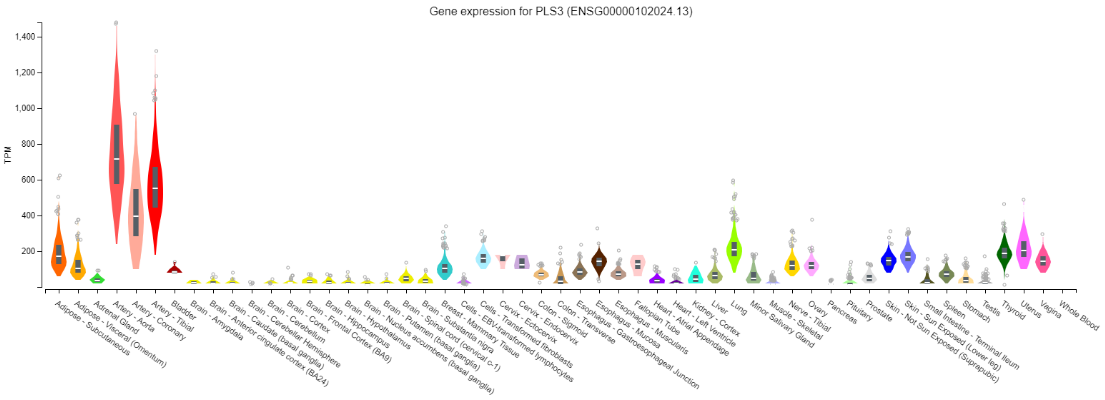

20. SEC24D

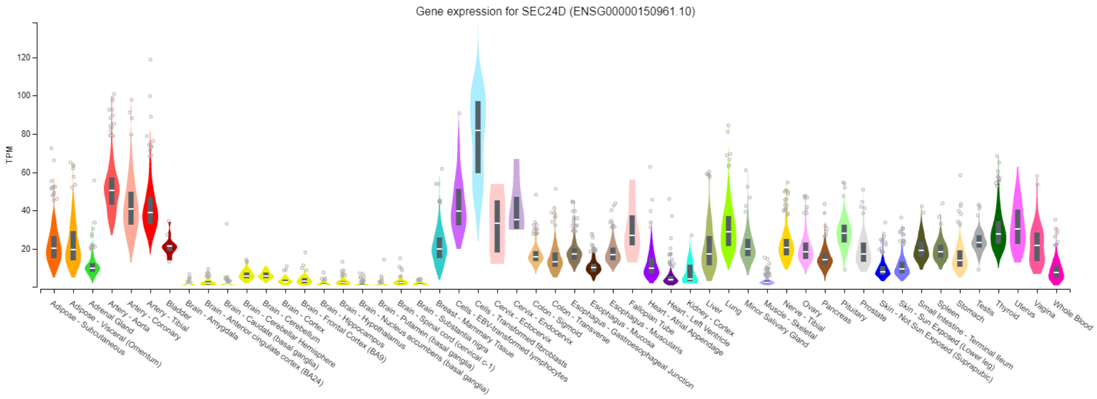

[**15 predicted genes**]:

1. ADAMTS2

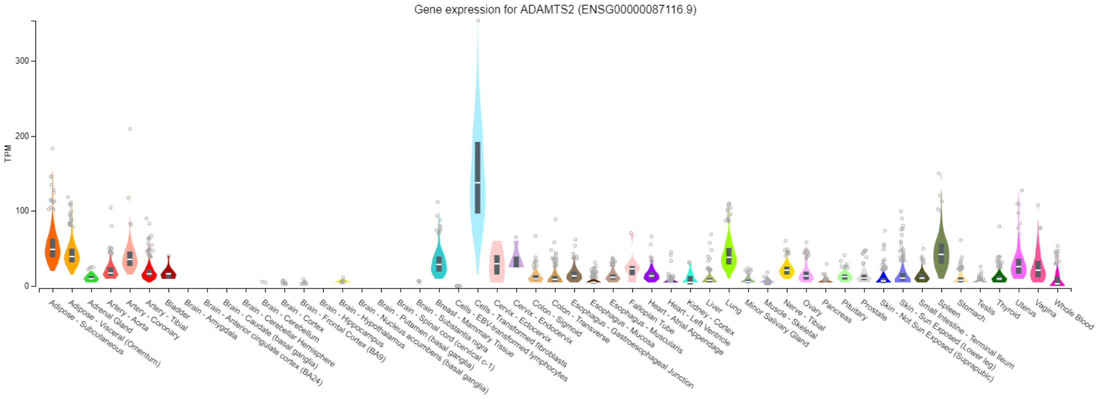

2. ADAMTS3

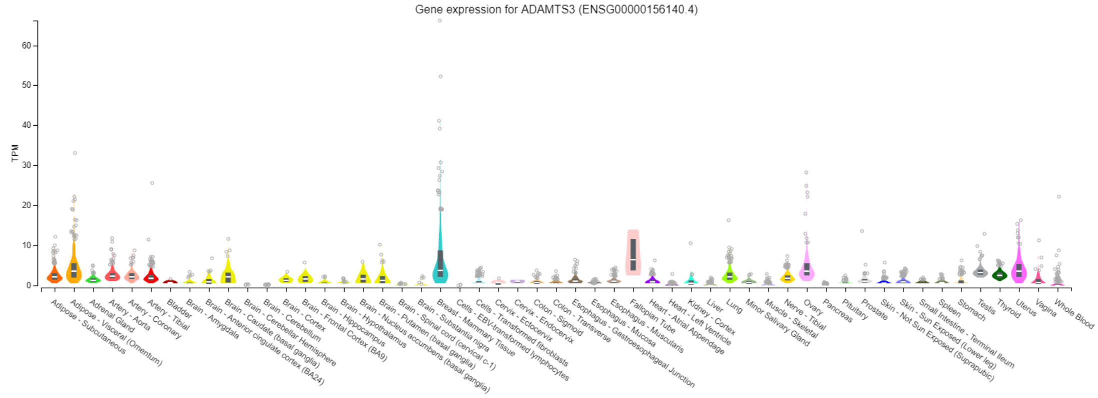

3. ADAMTS14

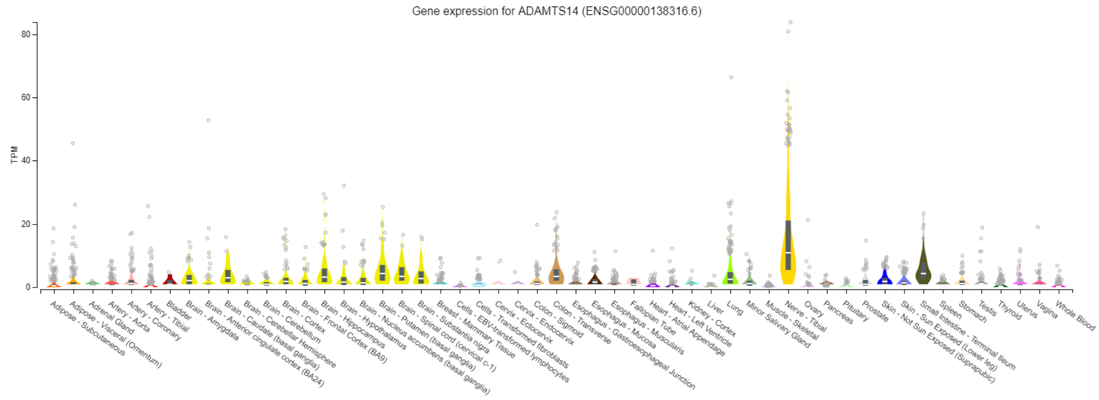

4. COL4A6

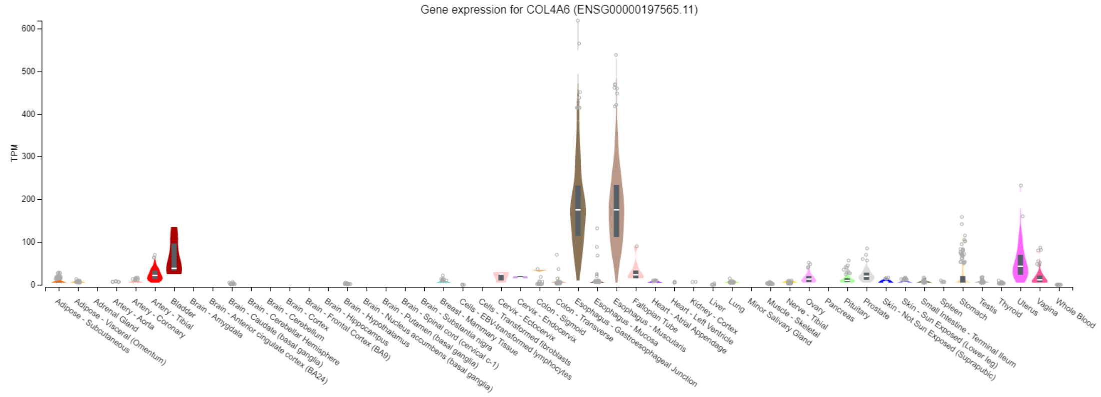

5. COL5A2

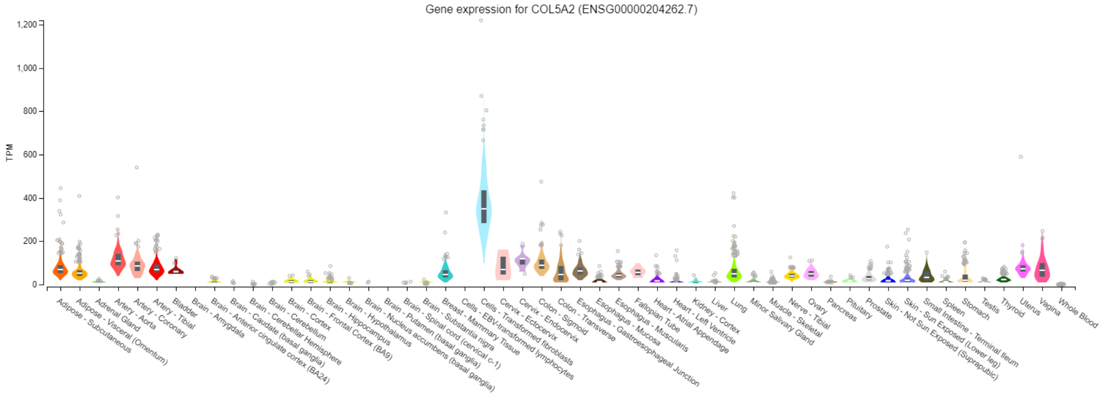

6. COL8A1

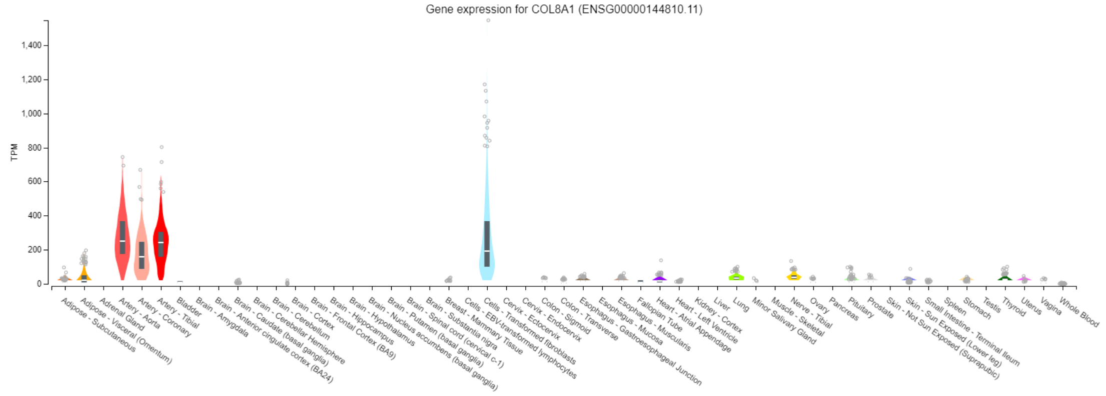

7. COL19A1

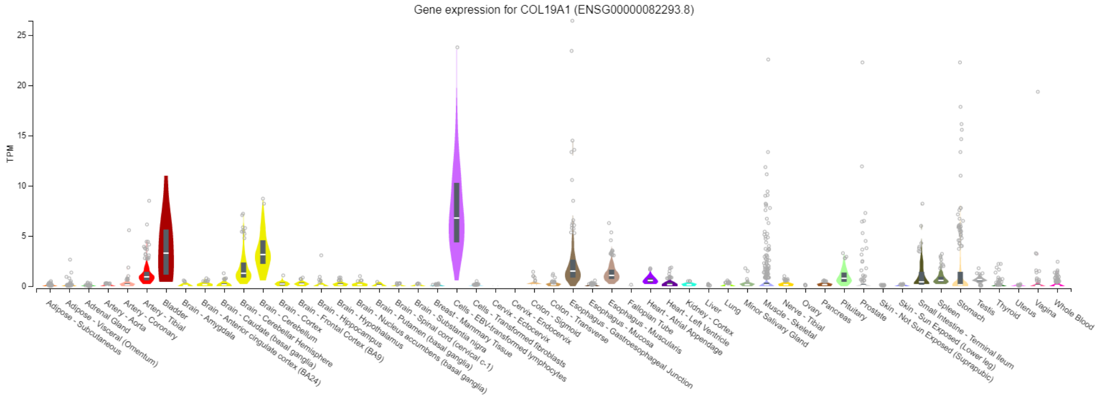

8. COL20A1

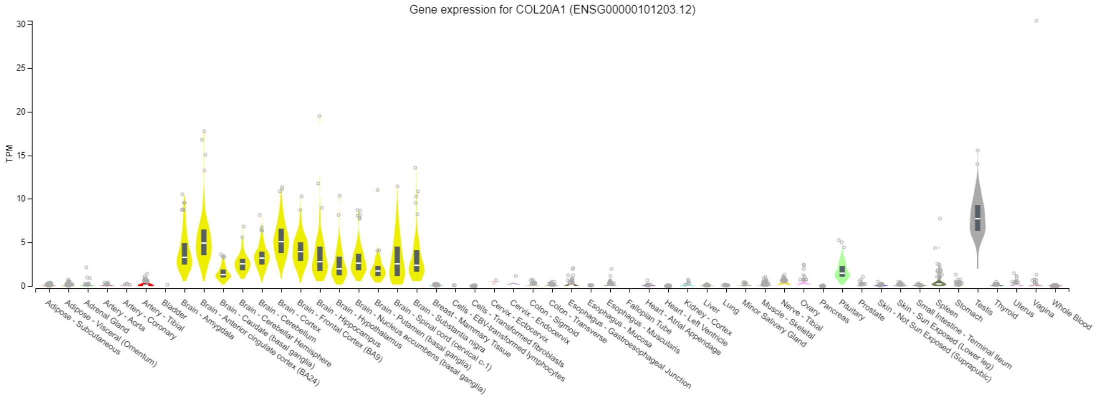

9. COL21A1

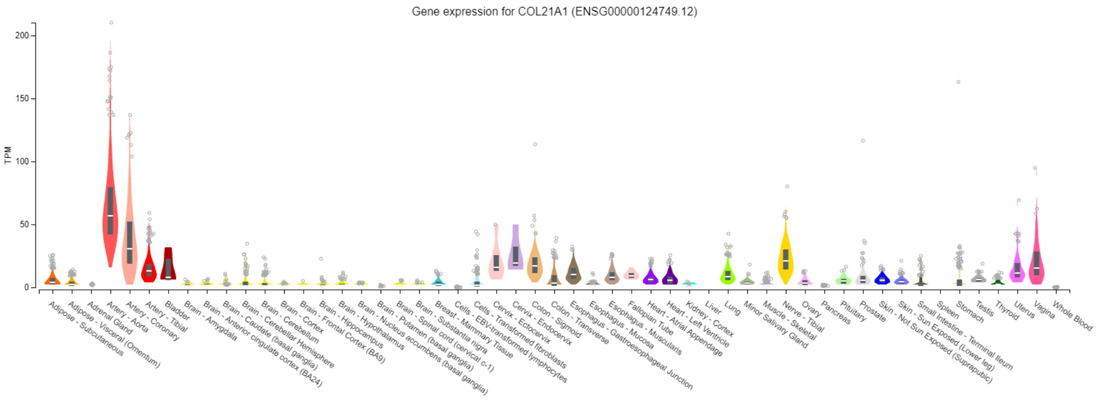

10. COL22A1

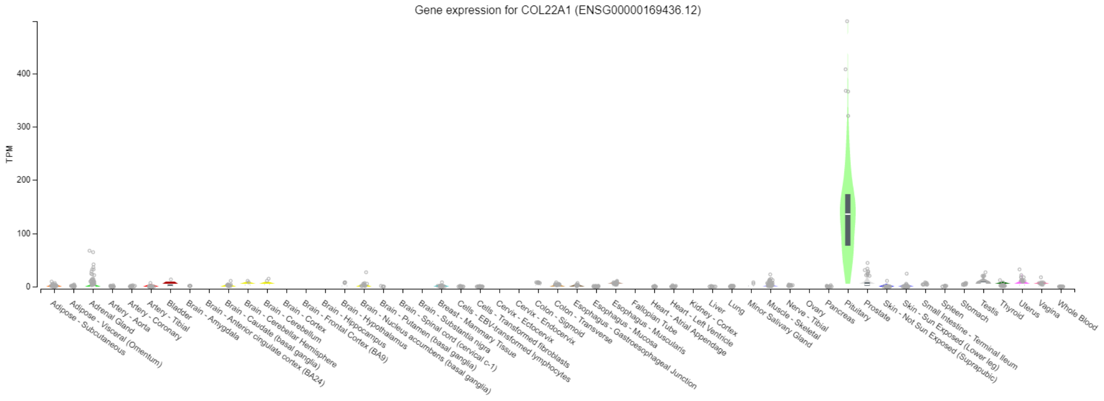

11. COL24A1

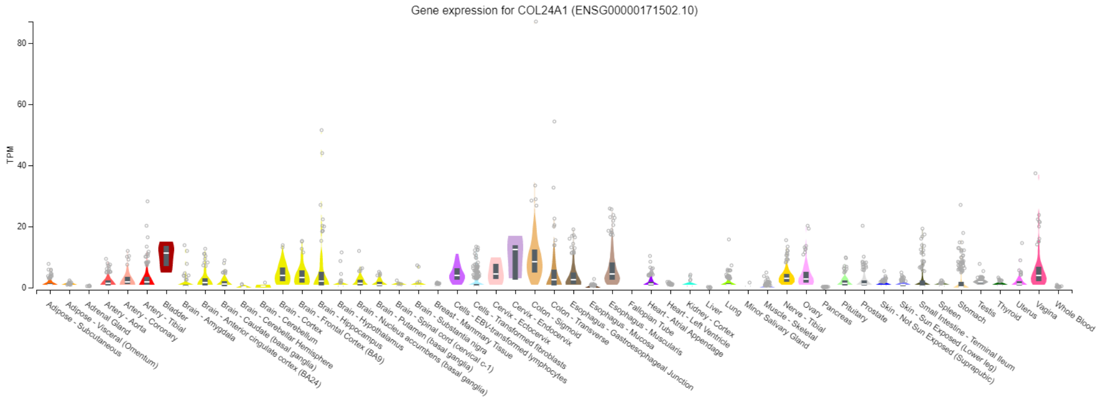

12. COL27A1

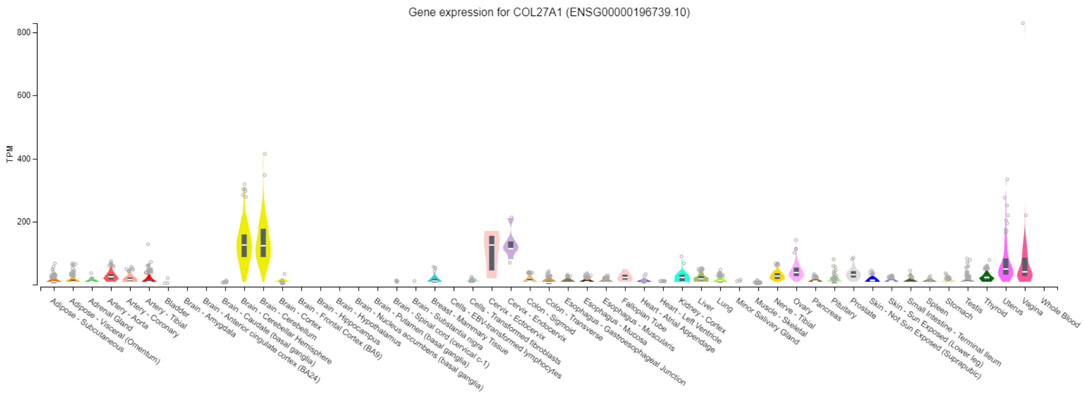

13. COL28A1

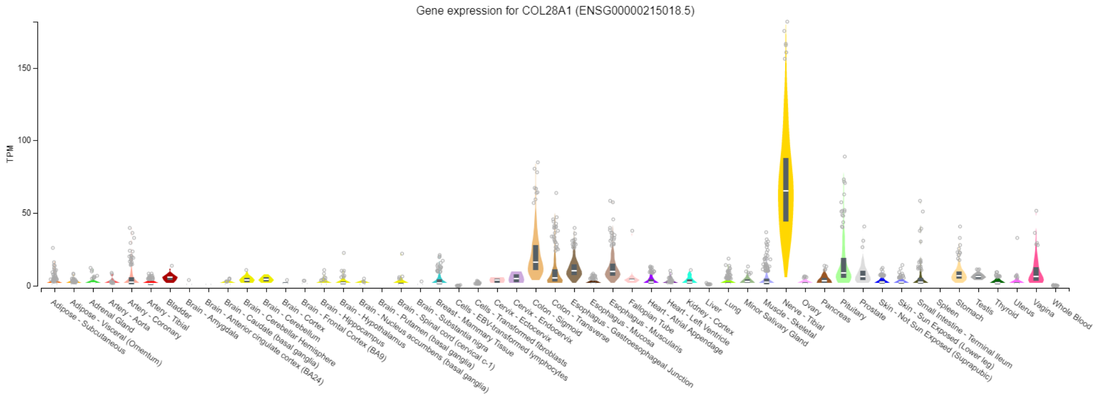

14. TLL1

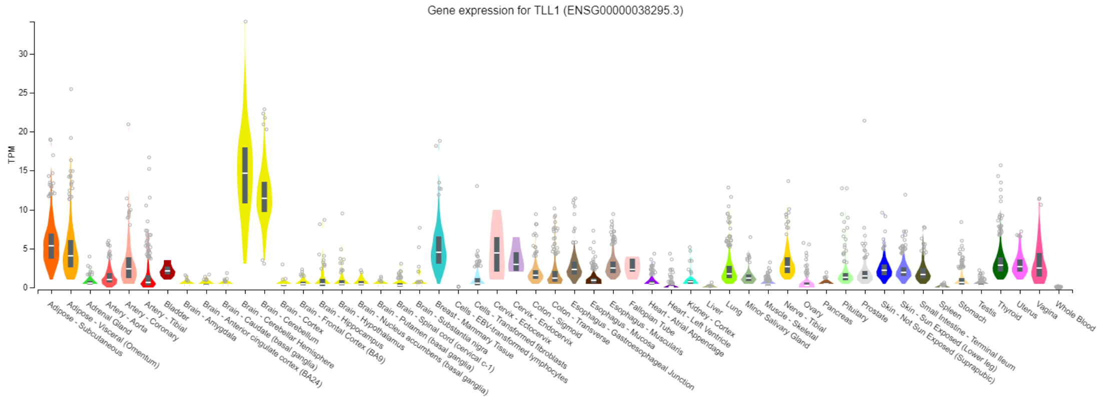

15. WNT8B

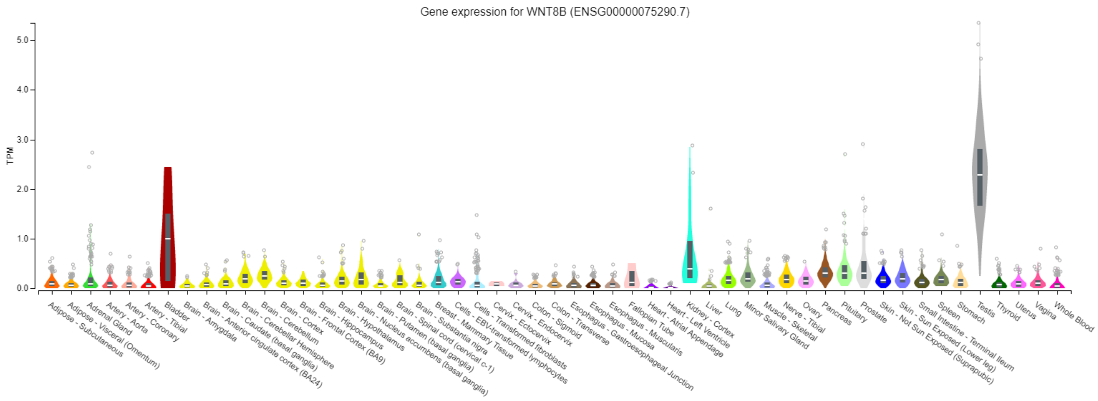

Supplement: Supplementary file 1 [file DataSheet_1.pdf]
